# Quantifying the dose-dependent impact of intracellular amyloid beta in a mathematical model of calcium regulation in xenopus oocyte

**DOI:** 10.1371/journal.pone.0246116

**Published:** 2021-01-28

**Authors:** Joseph Minicucci, Molly Alfond, Angelo Demuro, David Gerberry, Joe Latulippe

**Affiliations:** 1 Department of Chemistry and Biochemistry, Norwich University, Northfield, VT, United States of America; 2 Department of Mathematics, Norwich University, Northfield, VT, United States of America; 3 Department of Neurobiology and Behavior, University of California, Irvine, Irvine, CA, United States of America; 4 Department of Mathematics, Xavier University, Cincinnati, OH, United States of America; Georgia State University, UNITED STATES

## Abstract

Alzheimer’s disease (AD) is a devastating illness affecting over 40 million people worldwide. Intraneuronal rise of amyloid beta in its oligomeric forms (iA*β*Os), has been linked to the pathogenesis of AD by disrupting cytosolic Ca^2+^ homeostasis. However, the specific mechanisms of action are still under debate and intense effort is ongoing to improve our understanding of the crucial steps involved in the mechanisms of A*β*Os toxicity. We report the development of a mathematical model describing a proposed mechanism by which stimulation of Phospholipase C (PLC) by iA*β*O, triggers production of IP_3_ with consequent abnormal release of Ca^2+^ from the endoplasmic reticulum (ER) through activation of IP_3_ receptor (IP_3_R) Ca^2+^ channels. After validating the model using experimental data, we quantify the effects of intracellular rise in iA*β*Os on model solutions. Our model validates a dose-dependent influence of iA*β*Os on IP_3_-mediated Ca^2+^ signaling. We investigate Ca^2+^ signaling patterns for small and large iA*β*Os doses and study the role of various parameters on Ca^2+^ signals. Uncertainty quantification and partial rank correlation coefficients are used to better understand how the model behaves under various parameter regimes. Our model predicts that iA*β*O alter IP_3_R sensitivity to IP_3_ for large doses. Our analysis also shows that the upstream production of IP_3_ can influence A*β*-driven solution patterns in a dose-dependent manner. Model results illustrate and confirm the detrimental impact of iA*β*Os on IP_3_ signaling.

## 1 Introduction

Alzheimer’s disease (AD) is a devastating neurological illness affecting around 40 million people worldwide. AD is the leading cause of dementia, and while the prevalence is estimated to triple by 2050 [1], no cure currently exists. The progressive accumulation of intracellular A*β* in its soluble oligomeric forms iA*β*Os has been indicated as the leading event in the pathogenesis of AD [2–4]. A*β* is a 36-43 amino-acid-long peptide cleaved from the amyloid precursor protein (APP) by *β*- and *γ*-secretase. In neurons, cleavage of APP takes place when *γ*-secretase forms a complex with presenilin (PS) within the ER membrane, where production of A*β*_42_ is more likely to occur [5]. A*β* monomers tend to aggregate into soluble oligomers, fibrils, and plaques [6]. This aggregation occurs as the production of A*β* increases faster than can be degraded naturally [7, 8].

A*β* accumulation has been shown to occur as a result of multiple factors including overproduction of A*β* and aging-related changes in its clearance mechanisms; both by neuroglia and the lymphatic system [9, 10]. Importantly, the accumulation of intracellular A*β* has been shown to precede the appearance of extracellular amyloid plaques and intracellular neurofibrillar tangles associated with tau proteins, suggesting an early role of soluble A*β* during the progression of AD [7, 11–13]. The ability of extracellular applied A*β* oligomers to induce cytosolic Ca^2+^ fluxes generated from both extracellular and intracellular sources has been shown using cultured mammalian cells [14–16]. We have subsequently characterized these two mechanisms as occurring by: i) formation of plasma membrane Ca^2+^ permeable pores [17], and ii) permeation of A*β* oligomers into the cytosol and inducing a PLC-dependent Ca^2+^ release from the ER [18]. As a critical secondary messenger, Ca^2+^ mediates the signaling pathways that control several neuronal processes including neurotransmitter release, gene expression, metabolism, plasticity, development, proliferation, and cell death [19, 20]. Furthermore, accumulation of A*β* in neurons has been shown to disrupt intracellular Ca^2+^ homeostasis inducing mitochondrial stress [21, 22]. Because A*β* accumulation has been shown to alter intracellular Ca^2+^ levels, studying its impact on Ca^2+^ regulatory mechanisms is critical for better understanding the pathogenesis of AD.

Intracellular Ca^2+^ regulation involves many distinct mechanisms working together. In the presence of A*β*, these Ca^2+^ regulatory mechanisms begin to fail [22, 23]. For example, the presence of A*β* has been shown to increase Ca^2+^ liberation from the ER through 1,4,5-Inositol-triphosphate receptors (IP_3_Rs) and ryanodine receptors (RyRs) [15, 24]. A*β* can also spontaneously form Ca^2+^-permeable pores in the plasma membrane [20, 25] creating uncontrolled influx of Ca^2+^ through the membrane. These alterations can cause stress on the ER that can further lead to dysregulation of Ca^2+^ in a feed-forward cyclical pattern [15, 22, 26, 27]. Such breakdowns in regulation can create aberrant or sustained elevated Ca^2+^ signals that can lead to cell death [14, 18].

As A*β* has been shown to affect numerous intracellular pathways, it is difficult, if not impossible, for experimentalists to investigate independently and simultaneously each of these pathways in a complex neuronal environment. Mathematical and computational approaches can offer a supplementary approach to studying the pathology of AD and the impact of A*β* on cellular mechasims. Theoretical models that can consider the impact of A*β* on multiple pathways simultaneously and independently can provide valuable information for designing future experiments and possibly suggesting therapeutic targets. However, before such models can be constructed, developing dedicated models to investigate each proposed pathway involved in A*β* toxicity is crucial. To this point, our goal is to construct a data validated model that can quantify how A*β* interacts with the IP_3_ signaling cascade and its consequential disruption of intracellular Ca^2+^ homeostasis. Our single cell model provides important advantages toward the development of a whole-cell model, specifically allowing the study of A*β* in a cause and effect manner.

In our previous study, we have shown that intracellular injection of synthetic A*β*_42_ oligomers (A*β*_42_Os) into *Xenopus* oocytes triggered a PLC-dependent activation of IP_3_Rs in the ER membrane causing cytosolic Ca^2+^ rise [18]. However, experimental limitations make it difficult to precisely describe the molecular mechanisms involved. As such, we develop a mathematical model to identify and quantify the molecular mechanisms by which A*β* affects IP_3_ production and subsequent Ca^2+^ release through IP_3_Rs. We first build a computational model capable of tracking intracellular changes in Ca^2+^ concentration as a function of time. We assume that intracellular A*β*_42_Os (iA*β*_42_Os) have a direct impact on G protein activation and PLC-mediated IP_3_ production. The experimental results in [18] provide data to calibrate our mathematical model and to test our modeling assumptions. We show that increasing iA*β*_42_Os from small to large doses causes significant changes in the impact of A*β* on certain cellular mechanisms. Our model analysis substantiates that iA*β*_42_Os have a widespread effect on IP_3_-mediated Ca^2+^ signaling.

Because experimental recordings of Ca^2+^ signals are typically expressed as a ratio of fluorescence relative to the resting fluorescence before stimulation (Δ*f*/*f*_0_), we use the conversion methodology outlined in Maravall et al. (2000) [28] to directly compare our simulation results with experimental data. We further explore the implications of such conversion on model solutions and provide a detailed analysis of the impact of various model parameters along with predictions showing how the upstream mechanisms in IP_3_ production impacts Ca^2+^ signaling. Because model kinetics and parameters are linked to certain biophysical mechanisms, we use the model to study how changes in G protein and PLC activation rates impact Ca^2+^ signals. We also explore how large doses of iA*β*_42_Os alter the sensitivity of IP_3_Rs. Our results provide insight into which cellular mechanisms could become potential therapeutic targets for treating AD. Although A*β* can take many forms, in this work, we solely focus on iA*β*_42_Os, positively recognize by OC antibody and simply refer to them as A*β* for simplicity [6, 14].

## 2 Methods

### 2.1 The closed-cell model development

To investigate the impact of A*β* on Ca^2+^ regulation, we make use of the vast literature on calcium dynamics including the Ca^2+^ signaling “toolkit” [29–32]. We use experimental conditions and data from *Xenopus* oocytes to build a Ca^2+^ model using traditional methods of tracking the flux in and out of the cytoplasm. Let *c* denote the concentration of free Ca^2+^ ions in the cell cytoplasm, then the rate of change in intracellular Ca^2+^ can be modeled by

$$\begin{array}{c} \frac{dc}{dt}=J_{IN}-J_{OUT}, \end{array}$$

where *J* denotes flux across internal and external membranes.

While various pumps and channels exist between the ER and cytosol in neuronal and glial cells, intracellular Ca^2+^ signaling in *Xenopus* oocytes is mostly due to IP_3_Rs as oocytes are deficient in RyRs. In an *in vivo* environment, both the Na^+^/Ca^2+^ exchanger and the plasma membrane Ca^2+^ ATPase pumps affect Ca^2+^ removal from the cytosol while receptor-operated Ca^2+^ channels lead to Ca^2+^ entry into the cytosol from external sources. The experimental data on which we build the model are performed by monitoring the temporal evolution of the fluorescence signal generated by the bounding of cytosolic Ca^2+^ to the Ca^2+^-dependent fluorescent dye. As such, the data extracted from our experiments intrinsically take into account the endogenous activity of the Na^+^/Ca^2+^ exchanger, the plasma membrane and SERCA Ca^2+^ ATPase pumps in the absence of specific blockers.

Based on these conditions, we write

$$\begin{array}{c} \frac{dc}{dt}=J_{IPR}-J_{SERCA}+\alpha \left(J_{IN}-J_{PM}\right), \end{array}$$

where *J*_*IPR*_, *J*_*SERCA*_, *J*_*IN*_, and *J*_*PM*_ are the fluxes due to IP_3_Rs, SERCA pump, a plasma membrane channel (such as a Receptor Operated Channel), and Plasma Membrane pump, respectively. The constant *α* is typically used to control the rate of transport of Ca^2+^ across the membrane to that across the ER.

Let *c*_*e*_ denote the concentration of ER calcium. With this, we assume a Ca^2+^ model of the form

$$\begin{array}{ccc} \frac{dc}{dt} & = & J_{IPR}-J_{SERCA}+\alpha \left(J_{IN}-J_{PM}\right), \end{array}$$ (1)

$$\begin{array}{ccc} \frac{dc_{e}}{dt} & = & \gamma (J_{SERCA}-J_{IPR}), \end{array}$$ (2)

where *γ* is the ratio of cytoplasmic volume to ER volume. Note that we do not explicitly consider the effects of Ca^2+^ buffers. We assume that Ca^2+^ buffers are fast, immobile, and of low affinity (see [30, 32, 33] for further details on buffering). As such, Ca^2+^ buffering is implicitly included in the model by assuming that all fluxes are effective fluxes.

In our modeling analysis we assume that the contributions of *J*_*IN*_ and *J*_*PM*_ are small compared to the contributions of the ER. As such, we set *J*_*IN*_ − *J*_*PM*_ ≈ 0 and reduce the model to a closed-cell model where Ca^2+^ transport only occurs between the ER and cytosol. Understanding that stable Ca^2+^ oscillations in *Xenopus* oocytes occur in the absence of external Ca^2+^ suggests that Ca^2+^ exchange with the extracellular environment plays a minor role in the dynamics. However, this simplification does affect the biological implications and the model’s ability to describe Ca^2+^ regulation in general, and specifically in glial cells and neurons. For example, the direct exclusion of specific contributions from *J*_*IN*_ and *J*_*PM*_ may over-simplify Ca^2+^ solutions as the cell moves away from steady-state conditions. Furthermore, as cells are injected with A*β*, the contributions of the membrane transport mechanisms will certainly affect cytosolic Ca^2+^ concentration even in the absence of extracellular Ca^2+^. Regardless, the simplified deterministic model does allow us to illustrate important dynamical properties of Ca^2+^ signaling patterns with minimal components.

Accordingly, our closed-cell model assumes that Ca^2+^ flux into the cytosol is only due to the IP_3_R on the ER and flux out of the cytosol is due to an ATPase SERCA pump back into the ER. This simplified system allows us to model Ca^2+^ flux as a mean-field approximation process that considers an average over a large number of IP_3_Rs. While such a model can provide a macroscopic perspective across the whole cell, it cannot capture the stochastic nature of individual channel dynamics. However, such a model is appropriate for our goal of analyzing the influence of A*β* on the IP_3_ signaling cascade.

The flux terms in Eqs (1) and (2) can be modeled using various formulations, such as a saturating binding rate model for IP_3_R [34, 35] and Markov models [36–38]. For our purposes, we assume that the flux from IP_3_Rs follows a formulation based on previous models found in [39–41]. Thus, we write

$$\begin{array}{c} J_{IPR}=\left(k_{f}P_{o}+J_{ER}\right)\left(c_{e}-c\right), \end{array}$$ (3)

where *k*_*f*_ controls the density of IP_3_Rs, *J*_*ER*_ is the leak from the ER into the cytoplasm, and *P*_*o*_ is the open probability of the IP_3_R. In Eq (3), the leak term is necessary to balance the ATPase flux at steady state.

Recall that in our experiments, [18], individual cells were bathed in a Ca^2+^ free solution. As such, we assume a closed-cell environment with Ca^2+^ fluxes occurring only between the cytosol and the ER and set

$$\begin{array}{c} c_{t}=c+\frac{c_{e}}{\gamma }, \end{array}$$ (4)

where *c*_*t*_ is the total number of moles in the cell divided by the cytoplasmic volume [32]. We then replace the term (*c*_*e*_ − *c*) in Eq (3) with (*γ*(*c*_*t*_ − *c*) − *c*).

To model *P*_*o*_, we use the Li and Rinzel [42] simplification of the De Young and Keizer [39] formulation for the open probability of the IP_3_R

$$\begin{array}{c} P_{o}={\left(\frac{pc(1-y)}{\left(p+K_{1}\right)\left(c+K_{5}\right)}\right)}^{3}, \end{array}$$ (5)

where *y* is the proportion of inactivated IP_3_Rs and *p* is the concentration of IP_3_ present in the cytosol. To model the SERCA pump, we use a Hill function of degree two. Replacing the fluxes in Eqs (1) and (2), we have

$$\begin{array}{ccc} \frac{dc}{dt} & = & (k_{f}{\left(\frac{pc(1-y)}{\left(p+K_{1}\right)\left(c+K_{5}\right)}\right)}^{3}+J_{ER})\left(\gamma \left(c_{t}-c\right)-c\right)-\frac{V_{s}c^{2}}{K_{s}^{2}+c^{2}}, \end{array}$$ (6)

$$\begin{array}{ccc} \frac{dy}{dt} & = & [\frac{(k_{-4}K_{1}K_{2}+k_{-2}pK_{4})c}{K_{4}K_{2}\left(p+K_{1}\right)}]\left(1-y\right)-(\frac{k_{-2}p+k_{-4}K_{3}}{p+K_{3}})y, \end{array}$$ (7)

where *K*_*i*_, for *i* = 1, …5 and *k*_−4_ and *k*_−2_ are parameters associated with the transition rates between various quasi-steady-states of the IP_3_R (see [32, 42], and [30] for details), and *V*_*s*_ and *K*_*s*_ are the parameters associated with the SERCA pump.

**Fig 1:**
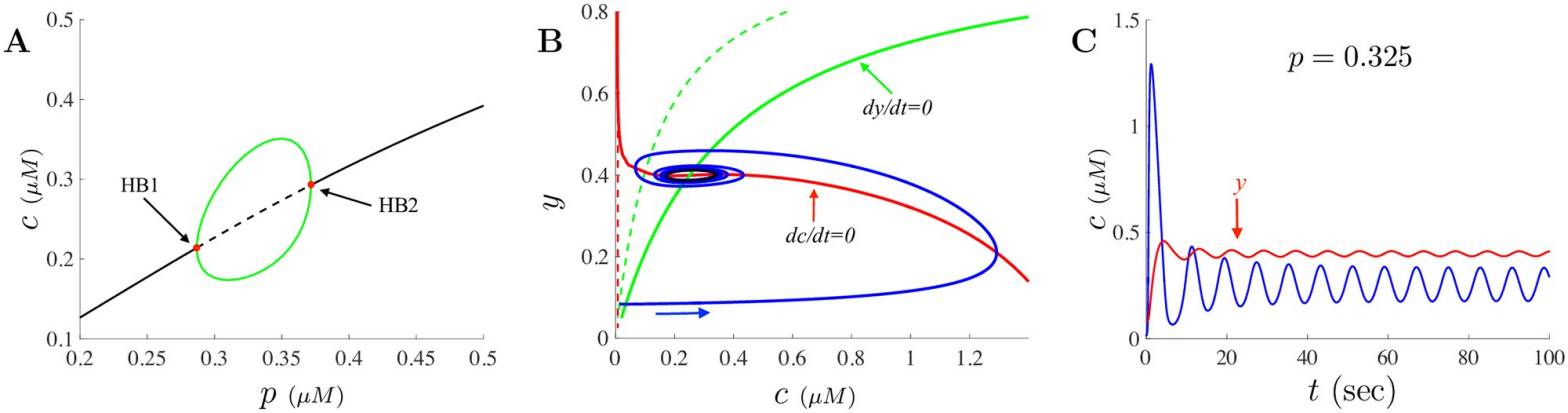
Dynamics and bifurcation structure for constant IP_3_. **A** shows the bifurcation structure for Eqs (6) and (7) for constant values of *p*. A classic Hopf bubble emerges between two Hopf bifurcation points labeled HB1 and HB2. The black solid line corresponds to stable fixed points while the dashed black curve are unstable fixed points. **B** shows the nullclines when *p* = 0 (dashed curves) and when *p* = 0.325 (solid curves). The nullclines corresponding to *dc*/*dt* = 0 and *dy*/*dt* = 0 are given by the red and green curves, respectively. A trace of the solution when *p* = 0.325 is given by the blue trajectory and is attracted to a period orbit (dark blue). The oscillating solution to the model when *p* = 0.325 is shown in **C** as a function of time with the corresponding solution to the *y* equation is shown in red.

The parameter values used for these equations are given in Table 1 and are similar to those used by De Young and Keizer [39] with modifications to the cellular and SERCA parameters. The choices for the the cellular and SERCA parameters were obtained by fitting the model to various experimental data illustrated in [18]. For these parameters, the dynamics of Eqs (6) and (7) are illustrated in Fig 1A where the steady-state values are shown as a function of *p*. As *p* increases, the dynamics illustrate the classic Hopf bubble and transitions from stable steady-states into periodic oscillations dynamics then back to stable steady-states through the Hopf bifurcation points labeled HB1 and HB2. The top and bottom branches of the bubble give the max and min values of the oscillations as a function of *p*. Shown in Fig 1B are the nullclines corresponding to *dc*/*dt* = 0 (in red) and *dy*/*dt* = 0 (in green) along with the trace of the solution when *p* = 0.325. The dashed lines correspond to the nullclines when *p* = 0 while the labeled solid red and green curves are the nullclines when *p* = 0.325. The temporal Ca^2+^ solution showing periodic oscillations when *p* = 0.325 is shown in Fig 1C. Also illustrated there is the variable *y* in red.

**Table 1:** Parameter values of the closed-cell Ca^2+^ base model. All IP_3_R parameters were adopted from De Young and Keizer (1992) while the Cellular and SERCA parameters were altered to match experimental results.

**Cellular Parameters**		**IP_3_ Receptor Parameters**	
*k*_*f*_	2.7 s^−1^	*K*_1_	0.13 *μ*M^−1^
*J*_*ER*_	0.00085 s^−1^	*K*_2_	1.05 *μ*M^−1^
*γ*	7	*K*_3_	0.943 *μ*M^−1^
*c*_*t*_	2 *μ*M	*K*_4_	0.145 *μ*M^−1^
		*K*_5_	0.082 *μ*M^−1^
**SERCA Parameters**		*k*_−2_	0.21 s^−1^
*V*_*s*_	1.5 *μ*M s^−1^	*k*_−4_	0.029 s^−1^
*K*_*s*_	0.15 *μ*M		

In [18], changes in Ca^2+^ concentration occur as a consequence of the intracellular injection of A*β*. As such, new IP_3_ is synthesized within the cell during the experimental procedure. To account for the IP_3_ dynamics, we use the hybrid formulation of Politi et al. [43]. Let *p* denote the concentration of IP_3_ present in the cytosol, then we write

$$\begin{array}{c} \tau_{p}\frac{dp}{dt}={\bar{V}}_{PLC}\frac{c^{2}}{K_{PLC}^{2}+c^{2}}-(\eta \frac{c^{2}}{K_{ip_{3}k}^{2}+c^{2}}+\left(1-\eta \right))p, \end{array}$$ (8)

where ${\bar{V}}_{PLC}$ is the maximal rate of IP_3_ production and depends on agonist concentration, *K*_*PLC*_ characterizes the sensitivity of PLC to Ca^2+^, *τ*_*p*_ = 1/(*k*_3*k*_+ *k*_5*p*_) represents the characteristic time of IP_3_ turnover where *k*_3*k*_ is the maximum rate of 3-kinase and *k*_5*p*_ is the dephosphorylation rate, *K*_*ip*_3_*k*_ is the half-activation constant for 3-kinase, and *η* = *k*_3*k*_/(*k*_3*k*_+ *k*_5*p*_). Both *K*_*ip*_3_*k*_ and *η* are used to tune the positive and negative feedback Ca^2+^ in the IP_3_ metabolism [30]. The term ${\bar{V}}_{PLC}$ will depend on the amount of activated PLC available and we alter the model by writing

$$\begin{array}{c} {\bar{V}}_{PLC}=V_{PLC}\cdot PLC, \end{array}$$ (9)

where *PLC* is the fraction of activated PLC complexes, and *V*_*PLC*_ is the IP_3_ maximal rate of production, to account for time evolving active *PLC*.

To model PLC and G-protein activation, we use a kinematic model due to Bennett et al. [44] and Lemon et al. (2003) [45]. We assume that *PLC* is the fraction of activated PLC complexes that drive IP_3_ production and that *G* is the fraction of activated G-protein complexes and write

$$\begin{array}{ccc} \frac{dPLC}{dt} & = & k_{a}G\left(PLC_{tot}-PLC\right)-k_{b}PLC, \end{array}$$ (10)

$$\begin{array}{ccc} \frac{dG}{dt} & = & k_{c}\left(\rho +\delta \right)\left(G_{tot}-G\right)-k_{d}G, \end{array}$$ (11)

where *PLC*_*tot*_ and *G*_*tot*_ are the total amount of available PLC and G-proteins (assumed fixed), *ρ* governs the production of active G-proteins, *δ* is used as a control for background activity, and *k*_*a*_, *k*_*b*_, *k*_*c*_, and *k*_*d*_ are rate constants. Notice that the kinetic formulations above are a simplification of the model constructed by Mahama and Linderman, [46], where a more complex set of equations that account for the hydrolysis of GTP to GDP. A summary of that model can be found in [30].

### 2.2 The effects of A*β*

Although A*β* is clearly implicated in the disruption of intracellular Ca^2+^ homeostasis, its interaction with individual pumps, channels, and exchangers remains difficult to quantify. In our previous experiments [18], we performed intracellular injections of A*β* oligomers at various concentrations levels. We also show that the injection of A*β* causes rise of cytotoxic levels of Ca^2+^ that carry on over time. This cytotoxicity may be due to stress caused by persistent Ca^2+^ release through IP_3_Rs. Of particular interest are the spatiotemporal patterns of fluorescence Ca^2+^ signals evoked by A*β* at dose of 1 *μ*g/ml. Recordings in different oocytes showed that A*β* led to various Ca^2+^ signaling with ranging patterns from slowly increasing to steady oscillations (Fig 1C-1E in [18]). Furthermore, when concentration levels of 3 *μ*g/ml, 10 *μ*g/ml, and 30 *μ*g/ml were utilized, the time courses of the fluorescence level of Ca^2+^ show that the amplitude of the Ca^2+^ signals increases, and the latency to onset and peak response time decreases as the amount of A*β* is increased [18]. In addition, the behavior of Ca^2+^ signals for the doses above 1 *μ*g/ml exhibited a prolonged time dependence with an increasing rapid decay as the amount of A*β* is increased. To capture the disparate Ca^2+^ signals evoked by various doses of A*β*, our model considers both “small” (1 *μ*g/ml or less) and “large” (greater than 1 *μ*g/ml) doses of A*β*. We utilize these results to hypothesize how A*β* impacts various cellular mechanisms in a dose-dependent manner, and how to incorporate A*β* into the model.

**Fig 2:**
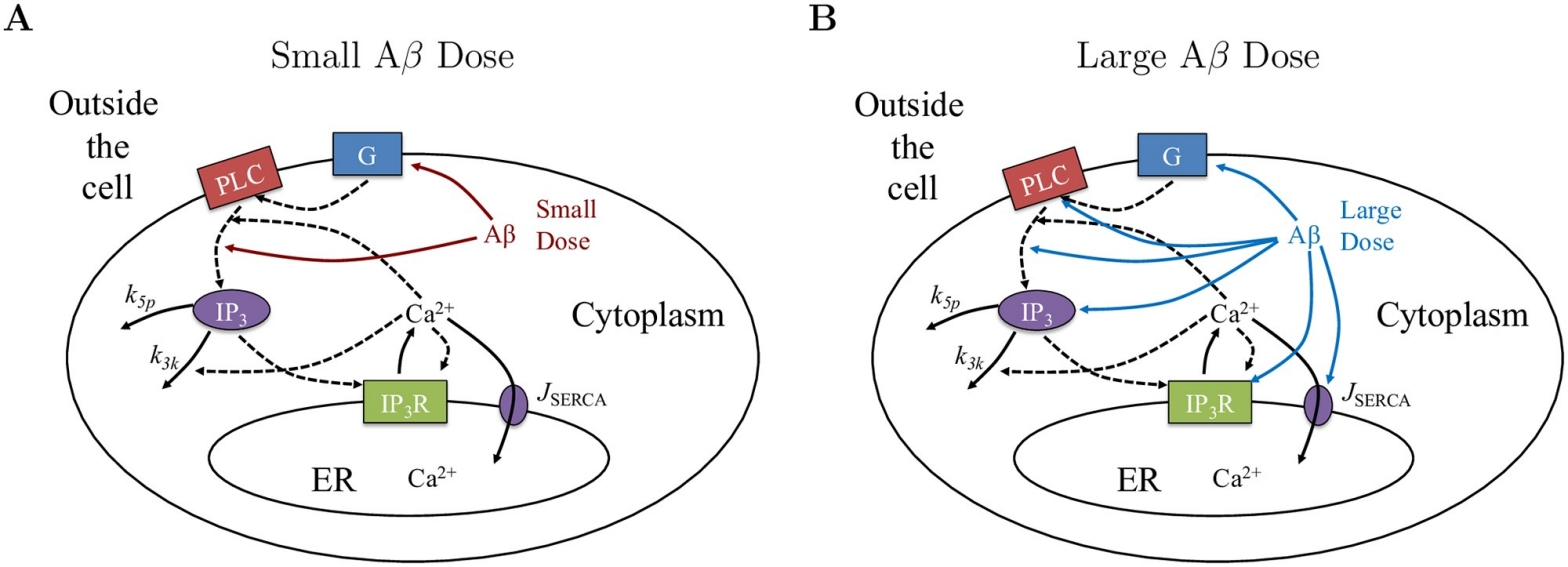
Model structure and components. Modeling assumptions for the location of impact of A*β* on the production of IP_3_ with key Ca^2+^ signaling mechanisms included in the closed-cell model. The key model assumptions for how A*β* impacts the IP_3_ signaling cascade are illustrated as red arrows for “small” doses in **A**. The impacted cellular mechanisms for “large” doses of A*β* are highlighted by the blue arrows in **B**.

Illustrated in Fig 2 are two diagrams showing the model assumptions for the interaction of A*β* on the IP_3_ signaling cascade along with the key model components for “small” and “large” doses. The black arrows (solid and dashed) emanating from and going into Ca^2+^ illustrate the flow of Ca^2+^ along with feedback mechanisms. The two red arrows emerging from A*β* in Fig 2A show the location of the impact of “small” doses of A*β* within the model structure. The blue arrows emerging from A*β* in Fig 2B show the mechanisms impacted by “large” doses of A*β*. The assumptions for how A*β* alters the mechanisms illustrated in Fig 2 are based on the model’s ability to reproduce dose-dependent experimental results and are discussed in greater detail below.

Our closed-cell model must be able to reproduce slow monotonic increases in Ca^2+^ as a result of the introduction of A*β*, as well as give rise to repetitive oscillations and baseline spikes for doses of 1*μ*g/ml. The model must also be able to reproduce and explain how A*β* leads to increasing signaling peak and a decreasing latency to peak of the response for “large” doses ranging from 3-30 *μ*g/ml. To determine the precise mechanisms by which A*β* affects the cellular machineries that regulate cytosolic Ca^2+^, using several antagonists, we suggest that A*β* acts upstream of IP_3_Rs and hypothesize that A*β* stimulates IP_3_ production by PLC in a G-protein-dependent manner [17]. Our modeling assumptions for incorporating A*β* were developed through a Monte Carlo Filtering process aimed to isolate the impact of A*β* within our model structure. First, we assume that A*β* acts as an agonist for G-protein activation and write

$$\begin{array}{c} \rho =V_{R}\cdot \frac{q}{K_{R}+q}, \end{array}$$ (12)

where *V*_*R*_ is a scalar, *K*_*R*_ is the A*β* concentration producing half activation. The term *q* represents the effects of a current injection at time *t* = *t*_1_ of A*β* at concentration *a* and has the form

$$\begin{array}{c} q=H\left(t-t_{1}\right)\cdot a\cdot e^{-r\left(t-t_{1}\right)H\left(t-t_{1}\right)}, \end{array}$$ (13)

where *H* is the Heaviside function and *e*^−*r*(*t*−*t*_1_)^ represents the decay of A*β* over time. To match the timeframe of the experimental injections, we set *t*_1_ = 2. In [18], A*β* responses were still evident after 10-15 minutes and as such, we assume a slow decay rate for A*β* and fix *r* = 0.001 in the model. In this representation, we are assuming that A*β* is acting like a G-protein agonist in a similar way as is expressed in [44].

Our second assumption is to alter the maximal rate of PLC mediated IP_3_ production to depend on A*β* as follows

$$\begin{array}{c} V_{PLC}=V_{0}+V_{Q}\cdot \frac{q^{2}}{K_{Q}^{2}+q^{2}}, \end{array}$$ (14)

where *V*_0_ accounts for PLC mediated IP_3_ production under normal conditions, *V*_*Q*_ accounts for influence of A*β* on PLC-mediated IP_3_ production, and *K*_*Q*_ is the dissociation constant. The exponent in *V*_*PLC*_ corresponds to a Hill coefficient of 2. A key finding based on this model formulation is that in order to match experimental results, PLC activation needed to be tied to A*β* concentrations. This assumption was determined critical for altering the amplitude of Ca^2+^ signals in coordination with the time to peak in our filtering process. Various alternative structures for *V*_*PLC*_ were explored numerically but those structures were deemed insufficient for generating the experimental Ca^2+^ signaling patterns outlined in [18]. As such, we have assumed that the maximal rate of PLC mediated IP_3_ production takes the form of Eq (14), but more data is needed to determine whether this assumption actually captures how A*β* alters *PLC*-mediated IP_3_ production.

Altogether, our closed cell model consists of five differential equations with A*β* input driving the system. In summary,

$$\begin{array}{ccc} \frac{dc}{dt} & = & (k_{f}{\left(\frac{pc(1-y)}{\left(p+K_{1}\right)\left(c+K_{5}\right)}\right)}^{3}+J_{ER})\left(\gamma \left(c_{t}-c\right)-c\right)-\frac{v_{p}c^{2}}{k_{p}^{2}+c^{2}}, \end{array}$$ (15)

$$\begin{array}{ccc} \frac{dy}{dt} & = & [\frac{(k_{-4}K_{1}K_{2}+k_{-2}pK_{4})c}{K_{4}K_{2}\left(p+K_{1}\right)}]\left(1-y\right)-(\frac{k_{-2}p+k_{-4}K_{3}}{p+K_{3}})y, \end{array}$$ (16)

$$\begin{array}{ccc} \tau_{p}\frac{dp}{dt} & = & (V_{0}+V_{Q}\frac{q^{2}}{K_{Q}^{2}+q^{2}})PLC(\frac{c^{2}}{K_{PLC}^{2}+c^{2}})-(\eta \frac{c^{2}}{K_{ip_{3}k}^{2}+c^{2}}+\left(1-\eta \right))p,\;\; \end{array}$$ (17)

$$\begin{array}{ccc} \frac{dPLC}{dt} & = & k_{a}G\left(PLC_{tot}-PLC\right)-k_{b}PLC, \end{array}$$ (18)

$$\begin{array}{ccc} \frac{dG}{dt} & = & k_{c}(V_{R}\frac{q}{K_{R}+q}+\delta )\left(G_{tot}-G\right)-k_{d}G, \end{array}$$ (19)

where the term *q* given in Eq (13) simulates the intracellular injection at time *t* = *t*_1_ of A*β* at concentration *a*. Base parameters for the IP_3_, PLC, and G-protein equations are given in Table 2. The parameters are separated by the dose of A*β* used in the model. We characterize a dose of 1 *μ*g/ml and smaller as “small” and doses above 1 *μ*g/ml “large”. We explain the distinction and need to separate the parameter space based on A*β* dosage below.

**Table 2:** Parameter values of the closed-cell Ca^2+^ model.

Model Parameters	Description	Small Doses (*a* ≤ 1)	Large Doses (*a* > 1)	Notes and Modeling Reference
**Cellular**				
*k*_*f*_	Maximal rate of Ca^2+^ release	2.7 s^−1^	3.5 s^−1^	Fit to experiment, [30, 32, 39]
*J*_*ER*_	ER Ca^2+^ leak	0.00085 s^−1^	0.0009 s^−1^	Fit to experiment, [30, 32, 39]
*γ*	Ratio of cytoplasmic to ER volume	7	8.5	Fit to experiment, [30, 32, 39]
*c*_*t*_	Total moles divided by cytoplasmic volume	2 *μ*M	2 *μ*M	[30, 32, 39]
**SERCA**				
*V*_*s*_	Maximal SERCA pump rate	1.5 *μ*M s^−1^	1.7 *μ*M s^−1^	Fit to experiment, [30, 32, 39]
*K*_*s*_	Half-activation SERCA constant	0.15 *μ*M	0.14 *μ*M	Fit to experiment, [30, 32, 39]
**IP_3_ Receptor**				
*K*_1_	IP_3_ receptor rate constant	0.13 *μ*M^−1^	0.21 *μ*M^−1^	[30, 32, 39]
*K*_2_	IP_3_ receptor rate constant	1.05 *μ*M^−1^	0.021 *μ*M^−1^	[30, 32, 39]
*K*_3_	IP_3_ receptor rate constant	0.943 *μ*M^−1^	0.943 *μ*M^−1^	[30, 32, 39]
*K*_4_	IP_3_ receptor rate constant	0.145 *μ*M^−1^	0.25 *μ*M^−1^	[30, 32, 39]
*K*_5_	IP_3_ receptor rate constant	0.082 *μ*M^−1^	0.01 *μ*M^−1^	[30, 32, 39]
*k*_−2_	IP_3_ receptor rate constant	0.21 s^−1^	0.012 s^−1^	[30, 32, 39]
*k*_−4_	IP_3_ receptor rate constant	0.029 s^−1^	0.00006 s^−1^	[30, 32, 39]
**IP_3_ Model**				
*V*_0_	Intrinsic PLC-mediated IP_3_ production	0.15 *μ*M	0.19 *μ*M	Fit to experiment
*V*_*Q*_	Control parameter for influence of A*β* on IP_3_	7.82 *μ*M	380 *μ*M	Fit to experiment
*K*_*Q*_	PLC dissociation constant	0.0086 *μ*g/ml	0.0086 *μ*g/ml	Fit to experiment
*K*_*ip*_3_*k*_	Half-activation for 3-kinase	0.6 *μ*M	1.6 *μ*M	Fit to experiment, [43]
*K*_*PLC*_	PLC sensitivity to Ca^2+^	0.01 *μ*M	0.016 *μ*M	Fit to experiment, [43]
*k*_3*k*_	IP_3_ phosphorylation rate	1.5 s^−1^	0.7 s^−1^	Fit to experiment, [43]
*k*_5*p*_	IP_3_ dephosphorylation rate	0.01 s^−1^	0.005 s^−1^	Fit to experiment, [43]
**PLC**				
*k*_*a*_	PLC-protein activation rate	0.35 s^−1^	0.75 s^−1^	Fit to experiment
*k*_*b*_	PLC-protein deactivation rate	2.2 s^−1^	2 s^−1^	Fit to experiment
*PLC*_*tot*_	Scaled total number of PLC	1	1	Fit to experiment
**G-Protein**				
*k*_*c*_	G-protein activation rate	0.33 s^−1^	0.047 s^−1^	Fit to experiment, [44–46]
*k*_*d*_	G-protein deactivation rate	2.17 s^−1^	4.7 s^−1^	Fit to experiment, [44–46]
*δ*	G-protein intrinsic activity	0.01	0.012	Fit to experiment, [44, 45]
*V*_*R*_	Maximal G-protein activation	7.4	10	Fit to experiment, [44, 45]
*K*_*R*_	A*β* concentration producing half-activation	4467 *μ*g/mL	2000 *μ*g/mL	Fit to experiment, [44, 45]
*G*_*tot*_	Scaled total number of G-protein	1	1	Fit to experiment, [44, 45]

## 3 Model results

### 3.1 Closed-cell model for small doses

In this section we investigate model solutions in relation to the experimental results described in [18] where a small amount of A*β* is used. A current injection of A*β* at dose of 1 *μ*g/ml gives rise to various spatio-temporal patterns in different cells ranging from a steady increase to periodic solutions. Although we are considering 1 *μ*g/ml a small dose, it was sufficient for evoking local puffs and global responses. Our ODE model cannot capture the traveling waves exhibited in the experiments, but we do show temporal Ca^2+^ oscillations that form the basis of wave activity. When the model given by Eqs (15)–(19) is simulated using the parameter values given in the Small Doses column of Table 2 with *a* = 1 *μ*g/ml of A*β*, we are able to reproduce many of the qualitative features illustrated in Fig 1 in [18]. For example, in some oocytes, a dose of 1 *μ*g/ml leads to a slow and steady increase in Ca^2+^ signals that persists. Other cells exhibit amplitude increasing oscillations or steady spike-like responses. These types of responses are captured by the model for baseline parameters with slight variation in the cellular parameters. Because Ca^2+^ recordings in Fig 1 in [18] come from different oocytes, we justify slight alterations to cellular parameters as a way to account for variations between individual cells. Note that IP_3_R parameters may also vary, but for now we simply focus on the SERCA parameter *K*_*s*_.

**Fig 3:**
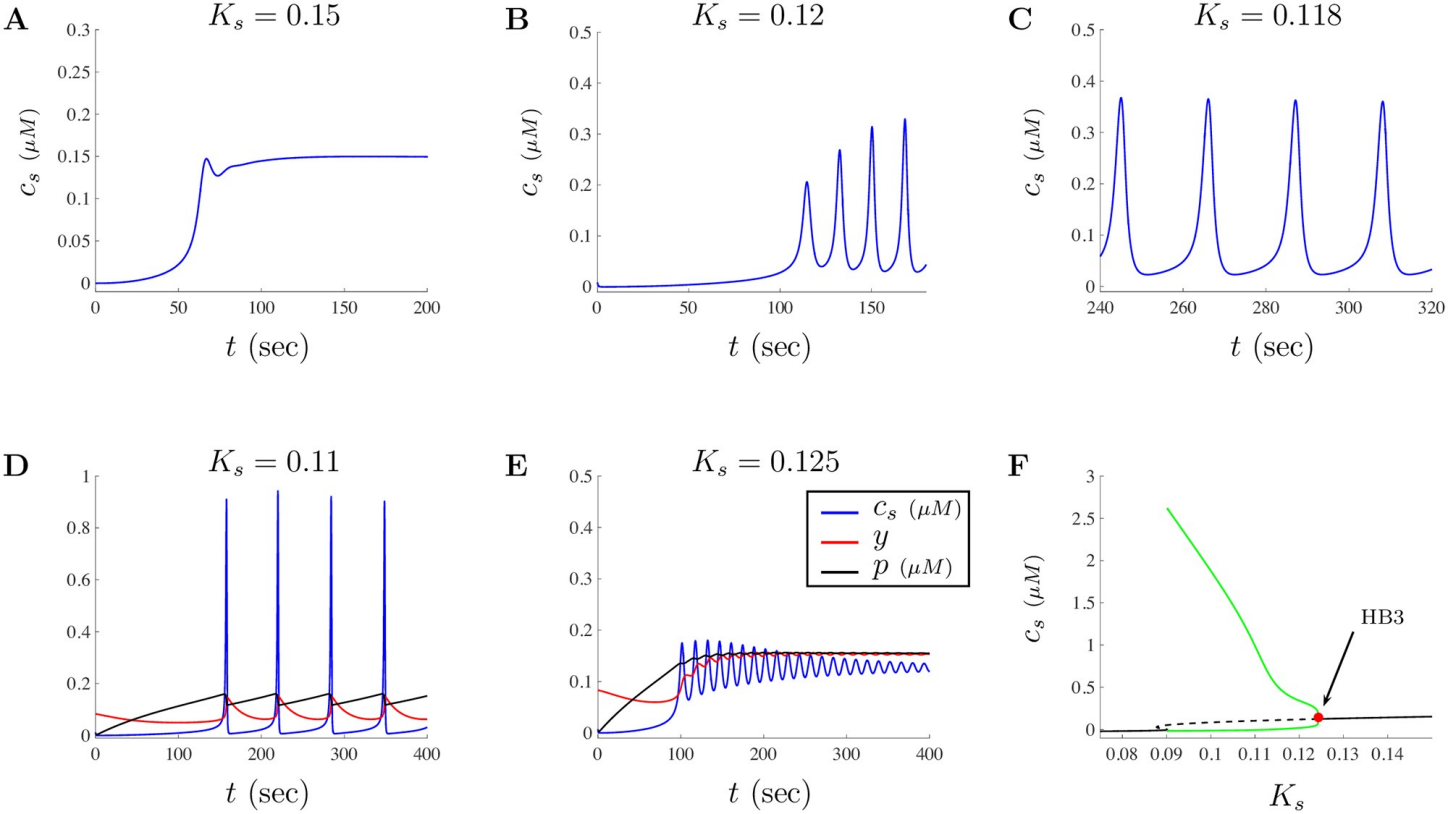
Model solutions mimic experimental Ca^2+^ patterns for doses of *a* = 1 *μ*g/ml of A*β*. The dependence of model solutions for a dose of 1 *μ*g/ml of A*β* on the cellular parameter *K*_*s*_ is investigated. **A** shows an increasing Ca^2+^ signal that settles to an increased steady-state when *K*_*s*_ = 0.15. **B** shows that oscillations in Ca^2+^ can exhibit increasing amplitudes such as those found in Fig 1D in [18]. **C** and **D** show that as the value of *K*_*s*_ decreases, the oscillatory patterns of the model reproduce the spike-like Ca^2+^ signals observed in Fig 1E in [18]. **E** illustrates an oscillatory solution with an increased steady-state Ca^2+^ homeostasis level when *K*_*s*_ is just above the Hopf point. Both **D** and **E** show the traces for *c*_*s*_, *y*, and *p* in blue, red, and black, respectively. **F** shows a simplification of the scaled bifurcation diagram with the bifurcation parameter *K*_*s*_. Notice that as *K*_*s*_ decreases from the base value of 0.15, a transition from stable steady-states into periodic oscillations occurs through a Hopf bifurcation around HB3≈0.1242. The dynamics around *K*_*s*_ = 0.09 include multiple Hopf bifurcations and has more complex structure than what is presented here.

Illustrated in Fig 3 are various scaled model solutions along with a partial bifurcation diagram highlighting the key behaviors of the model when *K*_*s*_ is altered. Model solutions illustrated in Fig 3 have been scaled according to the following

$$\begin{array}{c} c_{s}=\frac{c-c_{0}}{K_{D}}, \end{array}$$ (20)

where *K*_*D*_ = 0.3 is a dissociation constant that depends on indicator properties, and *c*_0_ is the resting Ca^2+^ concentration. In addition, to set the initial condition *c*_0_ (and those of the other variables) we first calculate the steady-state value for the parameter set when *a* = 0. As such, initial conditions for each of the solutions shown in Fig 3 are slightly different as altering *K*_*s*_ also changes the Ca^2+^ homeostasis level in the model but typically range between (0.01, 0.15).

Fig 3A–3C show responses similar to those in Fig 1 of [18]. More specifically, Fig 3A shows a solution where Ca^2+^ increases to a new steady-state when *K*_*s*_ = 0.15. Fig 3B illustrates a solution that has increasing amplitude oscillations when *K*_*s*_ = 0.12 while Fig 3C shows repetitive oscillations when *K*_*s*_ = 0.118. In both Fig 3B and 3C, model Ca^2+^ signals occur between 2-5 minutes, matching the experimental timescale for these types of responses. The responses in Fig 3D–3E show spike-like Ca^2+^ pattern that have a smaller frequency when *K*_*s*_ = 0.11 and a decreasing amplitude oscillatory solution when *K*_*s*_ = 0.125, respectively. A partial bifurcation diagram with *K*_*s*_ as the bifurcation parameter is provided in Fig 3F. As the parameter *K*_*s*_ decreases from *K*_*s*_ = 0.15, a transition from stable fixed points into periodic orbits occurs through a Hopf bifurcation point around HB3 ≈ 0.1242. As *K*_*s*_ continues to decrease, solutions will exhibit sustained oscillations with increased amplitude and a decrease in frequency. The dynamics of model solutions are much more intricate than the partial bifurcation diagram in Fig 3F suggests, especially around *K*_*s*_ = 0.09 where multiple limit points and Hopf bifurcations exist. However, our goal is not to fully examine the model dynamics but to merely show that by altering a single model parameter, we can generate solutions that are similar to experimental recordings. A complete description of the dynamics in this region is beyond the scope of investigation and is not included in our analysis.

*K*_*s*_ is the dissociation constant for the SERCA pump and is the Ca^2+^ concentration occupying half of the binding sites of the pump. A smaller *K*_*s*_ value corresponds to needing less Ca^2+^ to attain 50% of the maximal response for the pump. Whether changes in *K*_*s*_ are due to A*β* or simply through chance variation in cells remains debatable. Here, we argue that it is alterations in cellular structures modeled through differences in parameters that is causing the changes in Ca^2+^ signals and not because of A*β*’s direct impact on the SERCA pump. However, more analysis is needed to fully understand how different doses of A*β* may influence the generation of various Ca^2+^ signals.

**Fig 4:**
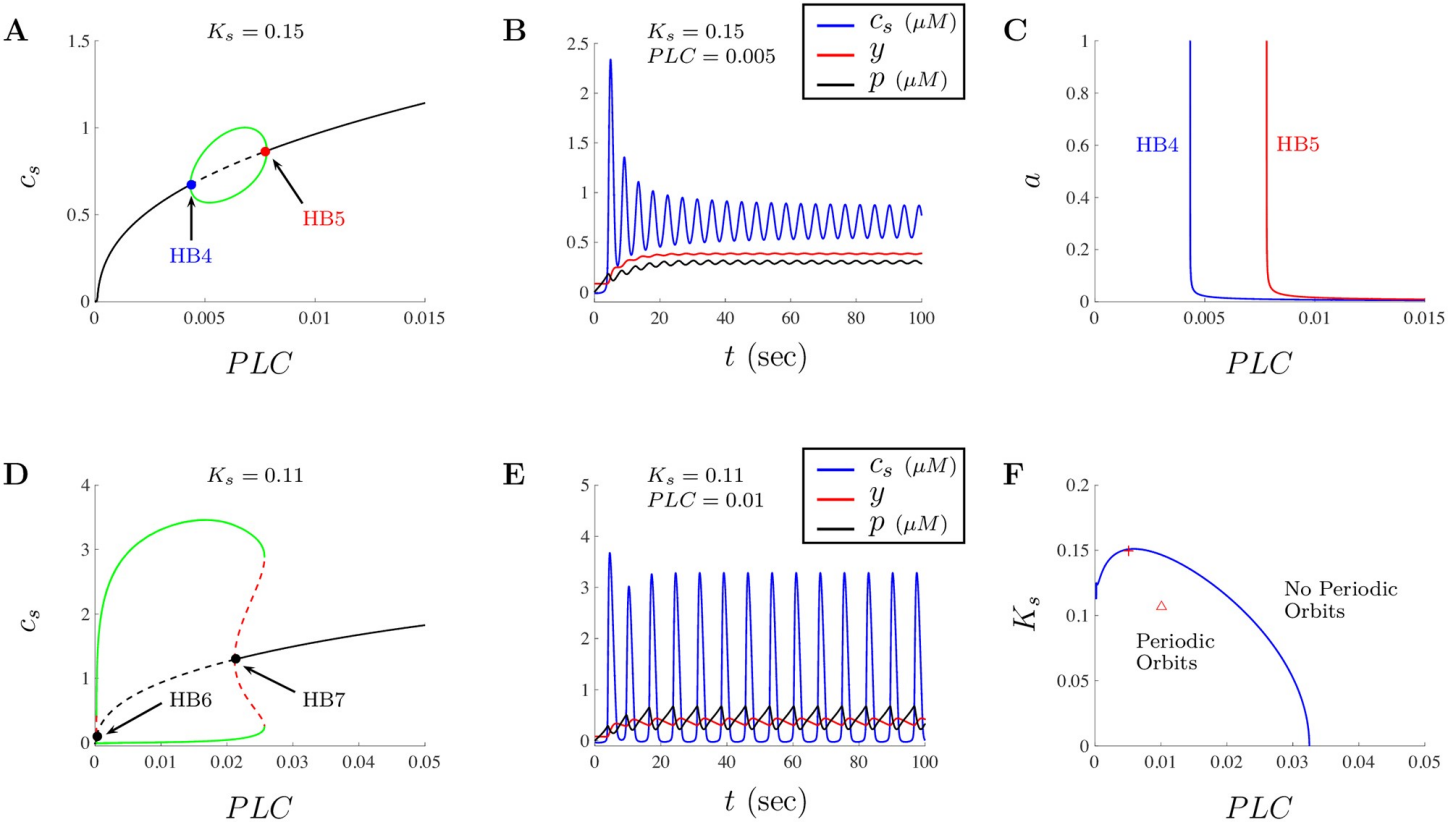
Dynamics of Ca^2+^ using *PLC* as a parameter for doses of *a* ≤ 1 *μ*g/ml of A*β*. Model dynamics for the subsystem Eqs (15)–(17) in terms of *PLC* and *K*_*s*_. The bifurcation diagram when *K*_*s*_ = 0.15 with the subsystem parameters given in Table 2, is shown in **A**. The figure shows a typical Hopf bubble between two Hopf bifurcation values labeled HB4 and HB5 as *PLC* is varied. The subsystem solution when *PLC* = 0.005 is presented in **B**, where *c*, *y*, and *p* are shown in the blue, red, and black traces, respectively. **C** shows the two parameter bifurcation diagram when both *PLC* and *a* are varied. Note that only the small doses of *a* are considered and the region of oscillations shifts to the right as *a* decreases and *PLC* increases. **D** shows the subsystem bifurcation diagram when *K*_*s*_ = 0.011 including two Hopf bifurcation values labeled HB6 and HB7. **E** shows the subsystem solution when *PLC* = 0.01 where *c*, *y*, and *p* are shown in the blue, red, and black traces, respectively. **F** shows the two parameter diagram tracking the location of the Hopf bifurcation points when *PLC* and *K*_*s*_ are treated as parameters. The parameter space is separated into regions where periodic orbits exist and don’t. The red cross and triangle correspond to the location of the parameter values used to generate the solutions in **B** and **E**, respectively.

When looking at the model, the subsystem given by Eqs (18) and (19) is driving IP_3_ through the inclusion of the *PLC* term in Eq (17). As such, we can investigate the subsystem given by Eqs (15)–(17) by treating *PLC* as a parameter and fixing *a* = 1. Illustrated in Fig 4A are the general dynamics of Ca^2+^ using *PLC* as a bifurcation parameter for the subsystem given in Eqs (15)–(17). Notice that the dynamics of Ca^2+^ will transition from stable steady-states (solid black curve), at the Hopf bifurcation point HB4≈0.0043 (labeled in blue), into periodic solutions until transitioning back to stable steady-states at Hopf bifurcation point HB5≈0.0078 (labeled in red). The green Hopf bubble captures the maximum and minimum values of the Ca^2+^ oscillations. Fig 4B shows the subsystem solution when *PLC* = 0.005 for the base parameters given in Table 2 for small doses. Intracellular Ca^2+^ signal, the proportion of inactivated IP_3_Rs, and the concentration of IP_3_ are given by the blue, red, and black traces, respectively. Since both *PLC* and *a* drive the responses of Eqs (15)–(17), a two parameter bifurcation diagram where the location of the Hopf points have been tracked as a function of *PLC* and *a* is given in Fig 4C. Notice that as the dose of A*β* gets closer to zero, the location of the Hopf bubble shifts to the right. This implies that in order to observe oscillatory behavior when *a* is close to zero, the amount of active *PLC* needs to be greater.

In Fig 3, we showed that the model solutions will behave differently as the cellular parameter *K*_*s*_ is varied. Here, we also look at the impact of varying *K*_*s*_ on the solutions of the subsystem Eqs (15)–(17). Illustrated in Fig 4D is the bifurcation diagram when *K*_*s*_ = 0.11. In this case, the bifurcation diagram shows an increased region of oscillations accompanied with increased amplitudes for most of the values of *PLC* in the range of the plot. Depending on the value of *PLC*, the oscillations will take more of a spiking form than sinusoidal oscillations, which is important as the signals observed experimentally correspond to spike-like signals of local puffs and global Ca^2+^ spikes. The red dashed curves in this figure correspond to unstable oscillations. The two Hopf bifurcations are given by HB6 ≈ 0.0002 and HB7 ≈ 0.02115. Fig 4E shows the subsystem solution when *PLC* = 0.01 and *K*_*s*_ = 0.11. To better understand the impact of changes in both *PLC* and *K*_*s*_ on the dynamics of the subsystem Eqs (15)–(17), a two parameter bifurcation diagram is given in Fig 4F. In this figure, the parameter space is separated into a region where periodic orbits exist and a region where the model has no periodic orbits. The blue curve in this figure tracks the location of the Hopf points generated by the subsystem. For values of *K*_*s*_ between approximately 0.1127 and 0.1511 the bifurcation diagram will have a Hopf-like bubble between two Hopf bifurcations (as those illustrated in Fig 4A and 4D). Although the complexities of these bifurcation diagrams varies, the two parameter bifurcation diagram helps us understand the oscillatory nature of solutions when variations in *PLC* and *K*_*s*_ occur. The red cross and triangle shown in Fig 4F correspond to the location of the parameter values for the diagrams generated in Fig 4A and 4B, and Fig 4C and 4D, respectively.

**Fig 5:**
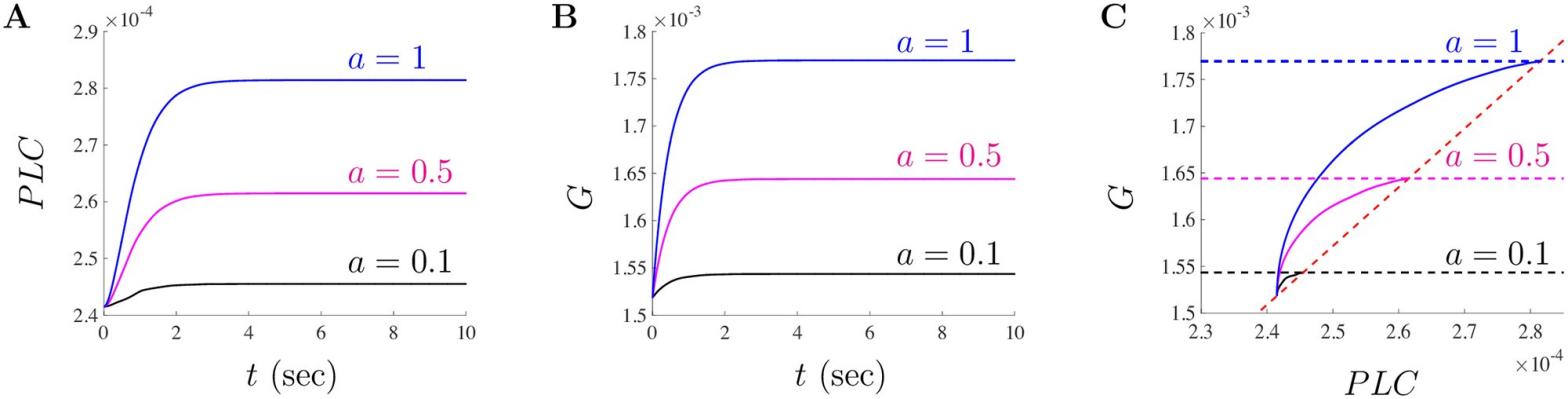
Steady-state values for *PLC* and *G* for doses of *a* ≤ 1 *μ*g/ml of A*β*. The steady-state fraction of activated PLC and G-proteins settles to a new value when *a* = 0.1 (black), *a* = 0.5 (magenta), and *a* = 1 (blue) in **A** and **B**, respectively. Model solutions for the various small doses *a*-values are shown on the phase plane for *PLC* and *G* in **C**. The dashed lines correspond to the *PLC* nullcline (red) and the *G* nullclines (black, magenta, and blue).

To further investigate the behavior of the small doses parameters, we decouple the *PLC* and *G* subsystem given by Eqs (18) and (19) and look at the time evolution of the fraction of active *PLC* and *G*. As *a* is varied for small doses, both *PLC* and *G* quickly (on the order of seconds) settle to their new steady-states values. Illustrated in Fig 5A and 5B are the temporal solutions of the subsystem (18) and (19) for *a* = 0.1 (black), *a* = 0.5 (magenta), and *a* = 1 (blue), respectively. Fig 5C shows the phase space solutions for *a* = 0.1 (black), *a* = 0.5 (magenta), and *a* = 1 (blue). The dashed lines in this figure correspond to the nullclines for Eq (18) (red) and Eq (19) (respective color). The new *a*-dependent steady-state values occur at the intersection of the respective dashed lines for each *a* value. All three solutions shown in Fig 5C start at the *a* = 0 equilibrium value of (*PLC*_0_, *G*_0_) ≈ (2.415 × 10^−4^, 1.518 × 10^−3^). When comparing the analysis shown in Fig 4, the values of PLC produced through the subsystem given by Eqs (18) and (19) will generate oscillatory responses when *K*_*s*_ is decreased from *K*_*s*_ = 0.15. Although additional analysis can be done for various parameters in the model, we now turn our attention to how altering the dose of A*β* impacts the model solutions.

### 3.2 Dose response relationship between amplitude and latency

**Fig 6:**
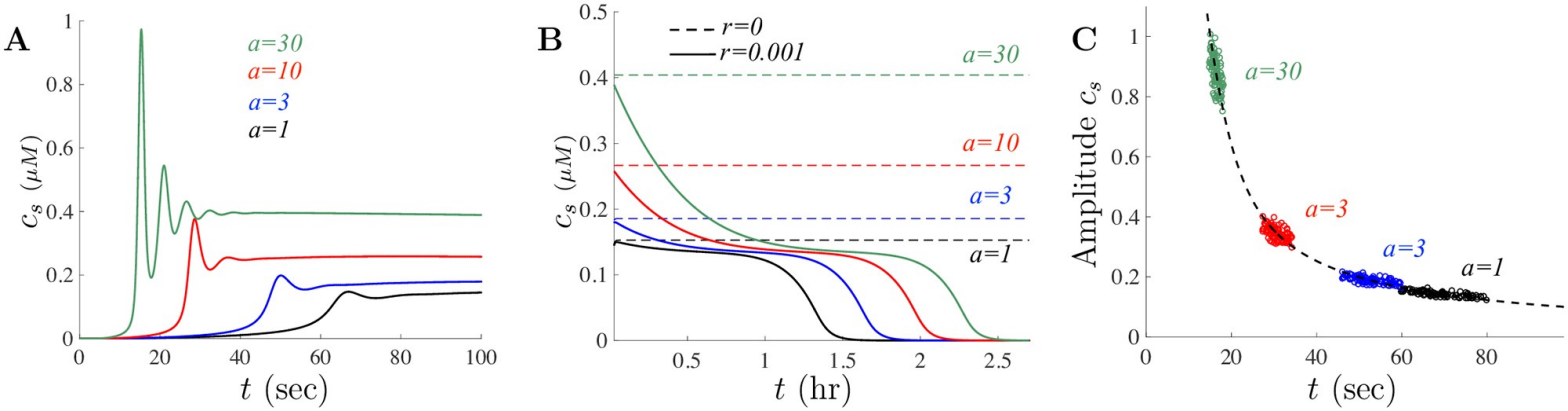
Amplitude and latency of model solutions vary with doses of A*β*. For the small doses parameters, **A** shows the model captures the increase in Ca^2+^ signal amplitude as well as the decrease in time to peak onset. **B** shows the long term impact of A*β* for doses of A*β* corresponding to *a* = 1, *a* = 3, *a* = 10, and *a* = 30 in black, blue, red, and green, respectively. The dashed lines in **B** correspond to the steady-state values in the event where the amount of A*β* does not decay and is fixed. **C** shows the location of 100 stochastically chosen cells under the given *a*-value. Each color-coded circle corresponds to the location of the solution peak and time of peak when cellular parameters are varied uniformly with 10% variation for the particular *a* value. The dashed black curve corresponds to the location of the amplitude peak for the small doses parameters for *a* ranging between (0.1, 40).

Dose-response experiments in *Xenopus* oocytes demonstrate two major effects on Ca^2+^ fluxes following increasing doses of A*β*: the amplitude of the Ca^2+^ signals increases with the amount of A*β* and the latency of the maximum peak time decreases as the amount of dose increases. We can test model against the experimental data starting with the small dose parameters to determine how the amplitude and latency of solutions vary as the doses of A*β* are increased. Illustrated in Fig 6A are scaled model solutions for A*β* doses of *a* = 1 *μ*g/ml (black), *a* = 3 *μ*g/ml (blue), *a* = 10 *μ*g/ml (red), and *a* = 30 *μ*g/ml (green) using the small doses parameters in Table 2. Notice that as *a* increases, the model captures both the amplitude increases and the decrease in latency to peak but is insufficient for reproducing the observed Ca^2+^ signals for large doses. Using the small dose parameters to study to explore model solutions and investigate the long term behavior of the model is helpful even though our analysis suggests needing two different dose-dependent parameter sets in order to match key experimental observations.

In the short term (on the order of minutes), solutions of the model with the small dose parameters tend to an apparent new homeostasis level. However, since the amount of A*β* introduced in the model through Eq (13) will eventually decay towards zero, the solution will tend back to the original steady-state value. This can be seen in Fig 6B where the model solutions are shown on a timescale of hours with *r* = 0.001 (the initial peak of solutions have been removed to better illustrate the long-term behavior). Whether A*β* decays naturally or persists in cells may depend on many factors. The Calcium hypothesis for AD suggests that the amyloidogenic pathway remodels the neuronal Ca^2+^ signaling pathway responsible for cognition [13, 47, 48]. As such, a slow accumulation of A*β* may increase the cytosolic Ca^2+^ level of cells leading to toxic stress and in turn can feed back into the hydrolysis of the amyloid precursor protein in a vicious cycle. In an *in vivo* environment, A*β* may slowly transition from small to large concentrations over timescales of months to years. Although any long-term analysis is beyond the current model, this model shows that if A*β* persists in the model (i.e., when *r* = 0), solutions would tend to new higher dose-dependent steady-state values as indicated by the dashed lines in Fig 6B. As expected, increasing *r* in the model will cause the solutions to decrease back to the original steady-state more rapidly.

To understand the impact of variations in the parameters on the amplitude and latency of solutions, Fig 6C shows the location of the solution peak as the parameters *k*_*f*_, *J*_*ER*_, and *γ* are uniformly varied 10% from base values for 100 trials. The dashed black curve in this figure corresponds to the amplitude and latency for the base small doses parameters in Table 2 for A*β* doses between *a* = 0.1 and *a* = 40. Notice that the amplitude is more variable than the latency for the dose of *a* = 30 while the opposite occurs for smaller values of *a*. Fig 6C is intended to illustrate that the model can capture some of the effects of “large” doses of A*β* as observed in Fig 1G of [18] while using the small doses parameters given in Table 2.

Interestingly, although the model given by Eqs (15)–(19) with the small doses parameters can capture many of the qualitative behaviors observed experimentally, it lacks some important features when large doses of A*β* are introduced. For example, the recorded average fluorescence response for doses of 3, 10, and 30 *μ*g/ml, have a much longer time dependence and display an increasingly rapid decay (see Fig 1G of [18]). These Ca^2+^ signals differ from responses evoked by a dose of 1 *μ*g/ml (such as those illustrated in Fig 3). The model solutions shown in Fig 6A do not capture these behaviors and as such cannot fully represent the impact of A*β* on cellular mechanisms (at least for large doses). Although we do not fully understand how large doses of A*β* affects the Ca^2+^ signaling cascade, our goal is to use the model to better understand how A*β* may be impacting individual cellular mechanisms through appropriate parameter selection. To do this, we alter model parameters to match the experimental data in Fig 1G of [18] and then use those results to describe the possible role that large doses of A*β* plays in Ca^2+^ signaling. In essence, in order to reproduce the observe experimental data when various doses of A*β* are used, we distinguish model behavior through the selection of small- and large-doses parameter sets.

## 4 Large doses parameter fitting

The model developed in the previous section tracks Ca^2+^ concentration as a function of time. The experimental data in [18] tracks changes in Ca^2+^ as a ratio of changes in fluorescence intensity with baseline fluorescence levels. This is often written as *δf* = (*f*−*f*_0_)/*f*_0_ = Δ*f*/*f*_0_ with *f*_0_ representing the fluorescence intensity at resting Ca^2+^ concentration. To better understand the impact of A*β* on Ca^2+^ dynamics through modeling, we first rescale fluorescence measurements to Ca^2+^ concentrations. According to Maravall et al. [28], changes in Ca^2+^ concentration are associated with changes in fluorescence through the equation

$$\begin{array}{c} c_{s}=f_{m}\left(1-1/R_{f}\right)\frac{\delta f}{\left(\delta f_{max}-\delta f\right)\delta f_{max}}, \end{array}$$ (21)

where *f*_*max*_ is the intensity of the dye at maximum Ca^2+^ concentration, *R*_*f*_ = *f*_*max*_/*f*_*min*_ is the indicator’s dynamic range with *f*_*min*_ being the intensity at minimum Ca^2+^ concentration, *δf*_*max*_ is the saturation of the Ca^2+^ indicator, and *f*_*m*_ = *f*_*max*_/*f*_0_. We use Eq (21) to rescale the experimental fluorescence data found in Fig 1G of [18]. Further details regarding the rescaling procedure are provided in the Appendix.

With the rescaling procedure described in the Appendix, we now have a way to convert the experimental fluorescence data in [18] to Ca^2+^ concentrations and link model solutions with experimental data. We first fix the scaling parameters *K*_*D*_ = 0.3, *R*_*f*_ = 100, *f*_*m*_ = 40 and then determine the value of the model parameters that will evoke the appropriate Ca^2+^ signals. The parameters used for the large doses of A*β* are given in Table 2 under the Large Doses column and were determined by fitting solutions to the converted experimental data for each level of A*β*. Starting with the small doses value, each parameter was stochastically chosen from an individual parameter distribution and a least-squares fitting procedure was used to identify a model parameter set corresponding to an approximate minimum of our objective function. We used a random sampling procedure to draw a parameter set *q*_*s*_ from an admissible parameter space $Q\in R^{p}$ (where *p* is the number of model parameters). The distribution of each parameter was chosen to match those of previous studies whenever possible. We then minimized the objective function

$$\begin{array}{c} Err=\sum_{i=1}^{n}{\left[sed\left(i\right)-ic_{s}\left(i,q_{s}\right)\right]}^{2}, \end{array}$$ (22)

where *sed*(*i*) is the scaled experimental data value at *i*, and *ic*_*s*_(*i*, *q*) is the corresponding (interpolated) scaled Ca^2+^ solution at *i*.

Our minimization technique uses a random sampling procedure with a random walk process when local minima are found. That is, we randomly select parameter values and compute *Err*. If *Err* is less than some threshold, we then perform a random walk around the parameter values that generated the local minimum error *Err* to locate a local minimizer. While minimizing the objective function for a large number of parameter selections provides potentially good estimates for model parameters, we did not analyze the parameter space with the intention of finding a global minimum. Regardless, the minimization technique does provide a way to establish parameter values that otherwise would be difficult to estimate.

**Fig 7:**
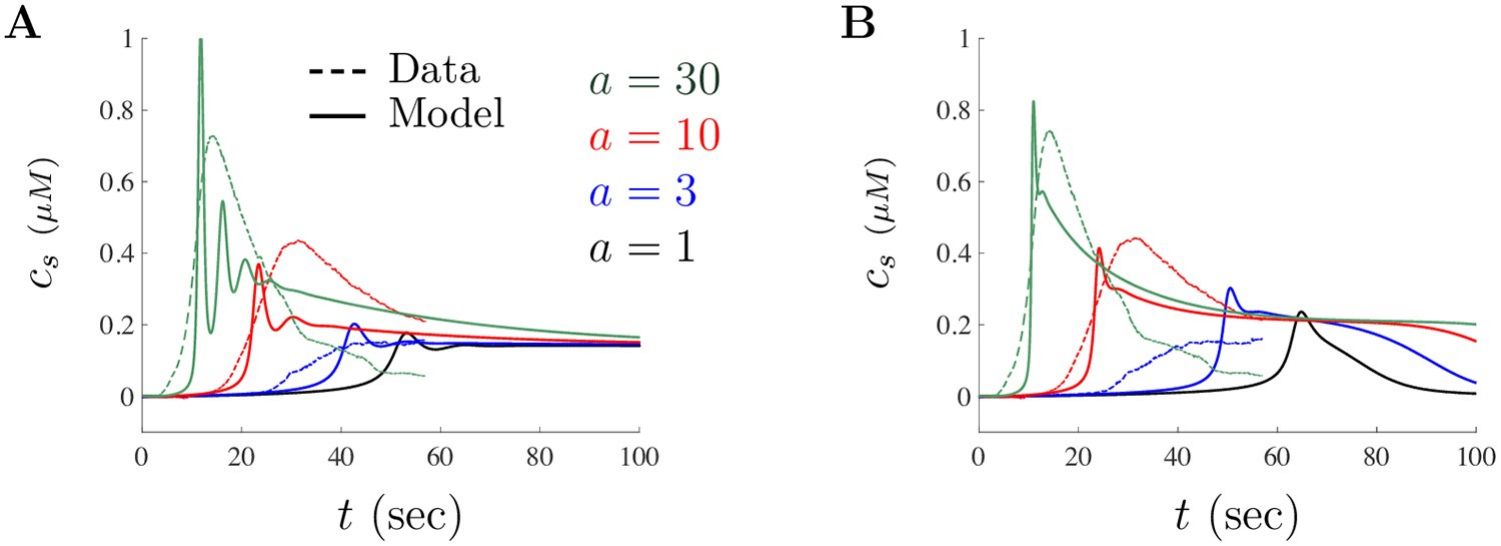
Variation in all parameter subsets required to reproduce the impact of A*β* for large doses. **A** shows a “best” fit solution when the Cellular, SERCA, and the IP_3_ Receptor parameters are kept fixed. Notice that altering the remaining parameters (IP_3_ Model, PLC, and G-Protein) cannot capture the observed Ca^2+^ signal. **B** shows a “best” fit solution when only the IP_3_R parameters are kept fixed. Notice that without altering the IP_3_R parameters, model solution peaks and decay also do not reproduce the observed experimental behaviors for large doses of A*β*.

To further understand the impact of parameters on model solutions, we also implemented an additional minimization technique where we took individual parameter subsets from Table 2, varied those, then compared the results with the experimental data. For example, starting with the small doses parameters, we only varied the *PLC* parameters to determine whether changes in those parameters could capture the large doses experimental results, and so on. This process was conducted for many parameter subset combinations starting with the small doses parameter set. Illustrated in Fig 7 are two “best fit” scaled model solutions *c*_*s*_ (smooth curve) shown on top of the scaled experimental data (dashed curve) for each of the three A*β* concentrations. Fig 7A shows a best fit solution when Cellular, SERCA, and the IP_3_R parameters are keep fixed. Observe that the “best” fit parameters are not those listed in Table 2 for either the small or large doses since we are varying some parameters and keeping others fixed. Note that the best fit solutions illustrated here are not much different from the solutions shown in Fig 6A. This suggests that alterations in some of the Cellular, SERCA, and IP_3_R parameters appear to be necessary to capture the observed behavior for large doses of A*β*. Similarly, Fig 7B shows a best fit solution when all but the IP_3_R parameters are varied. This simulation is included to clearly illustrate the need for altering all model parameters, particularly the IP_3_R parameters. The results of these simulations demonstrate that A*β* has a pervasive effect on the entire cell structure in large doses since matching the experimental data did require variation in every set of cellular mechanisms included in the model.

**Fig 8:**
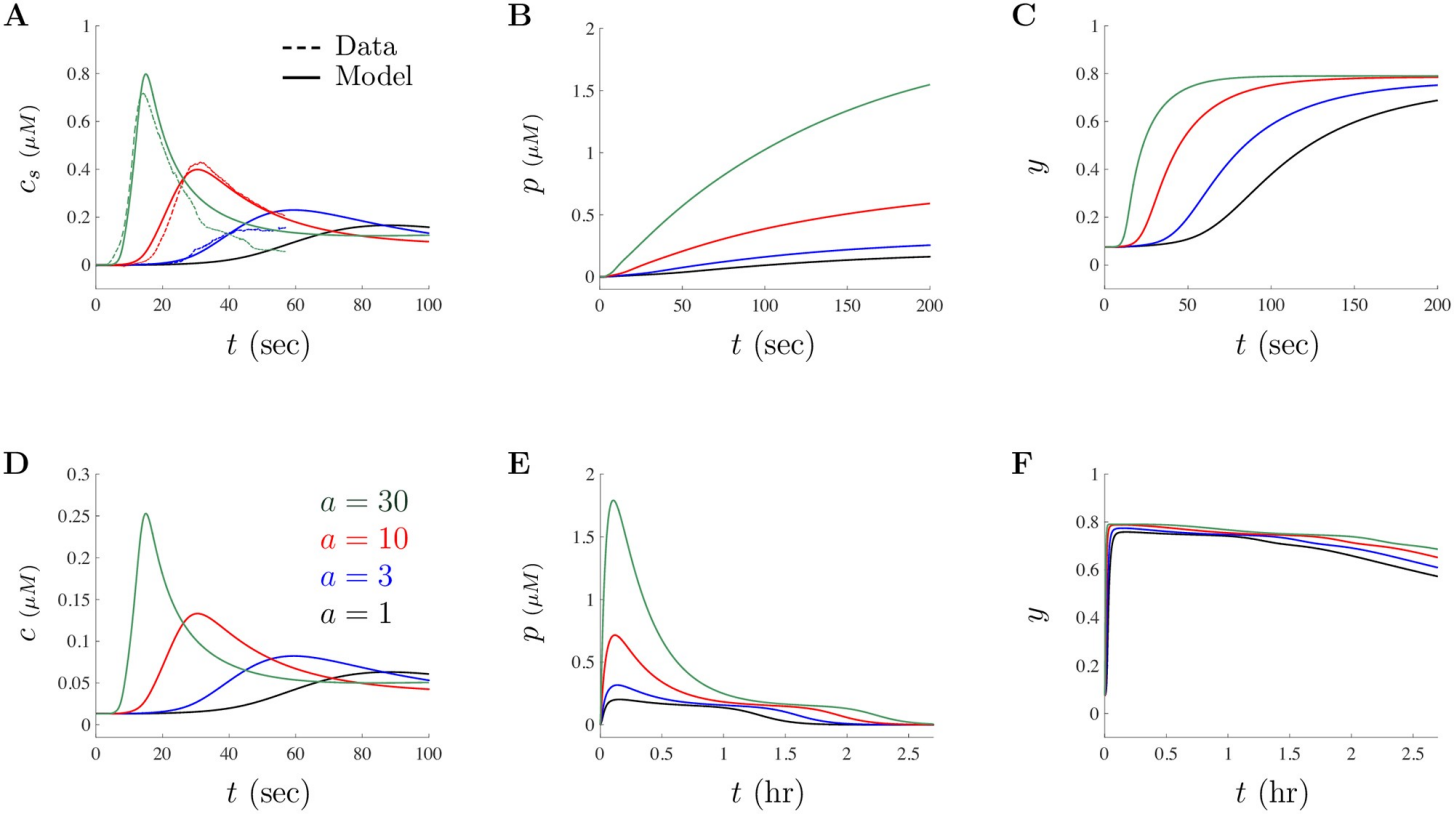
Model matches experimental data for large doses of A*β*. Model solutions for the “large” dose parameter set are illustrated in this figure. **A** shows model simulation (smooth curve) when *a* = 3 (blue), *a* = 10 (red), and *a* = 30 (green) overlaid on top of the rescaled experimental data (dashed curve) of [18]. Note that model solutions for the “large” dose parameters when *a* = 1 (black) is also shown here. **B** and **C** show the time evolution of model IP_3_ and PLC, respectively. **D** shows the unscaled Ca^2+^ concentration given by the model variable *c* for the three levels of A*β* and for *a* = 1 (black). **E** and **F** show the time evolution of *p* and *y* on the order of hours, respectively. Due to the A*β* decay incorporated in the model, all model solutions will eventually go back to the steady-state values.

Illustrated in Fig 8 are model solutions when *a* = 3, *a* = 10, and *a* = 30 using the large doses parameters given in Table 2. We also included the solution for *a* = 1 to illustrate how this large doses model behaves for the dose of 1 *μ*g/ml for comparison. Fig 8A shows the scaled model solution (smooth curve) on top of the scaled experimental data (dashed curve) for each of the three A*β* concentrations. Fig 8D shows the unscaled Ca^2+^ concentration *c* illustrating that the scaling procedure does not effect the model’s ability to capture the general behavior of the Ca^2+^ signals observed experimentally. Solutions for *p* are plotted in Fig 8B and 8E using two different timescales. Again, in our model A*β* decays exponentially and over the course of a couple of hours, the model solutions settle back to their original steady-states. Fig 8C and 8F show the evolution of *y* using two different timescales. These two figures show that the proportion of IP_3_Rs that are inactivated by Ca^2+^ remains fairly high over the course of hours acting to suppress Ca^2+^ spikes over time.

All model parameters used in the simulations illustrated in Fig 8 are given in Table 2 under the Large Doses column. Notice that the differences in each solution (as given by the different colors) of Fig 8A is only driven by changes in the value of *a*. In all simulations, initial conditions were found using the steady-state values when *a* = 0. Noteworthy, our large doses model is efficiently capable of capturing the increase in amplitude of the Ca^2+^ concentration signal and the decrease in latency to peak onset as well as increasingly rapid decay as the A*β* concentration *a* is increased, agreeing well with high suitability the experimental data for large doses of A*β*. Furthermore, the model with this parameter set is able to capture the slowly increasing Ca^2+^ response seen in some oocytes with a dose of 1 *μ*g/ml (such as responses similar to those shown in Fig 3A), but it cannot reproduce the various oscillatory and spiking behavior through small variations in parameters (such as those shown in Fig 3B–3E). The model with the small doses parameters cannot capture the increasingly rapid decay based on A*β* nor the extended time dependence, underscoring the need for two different parameter sets. The difference in parameter values between the two sets suggests that A*β* has a pervasive impact that permeates throughout a cell over time and gives credence that A*β* may indeed be affecting multiple cellular mechanisms simultaneously.

### 4.1 Uncertainty quantification and partial rank coefficient correlation for large doses

As with any experimental procedure, uncertainty in measurement naturally arises within the environment. These variations mean that finding exact values for model parameters is unrealistic. Performing uncertainty quantification allows us to determine how changes in parameter inputs affect model solutions. For example, in [18] Ca^2+^ responses are categorized by the change in fluorescent signaling and results are given as an average of 4-5 cells. Responses from individual cells can also change from cell to cell and as such, there could be natural variations in output.

**Fig 9:**
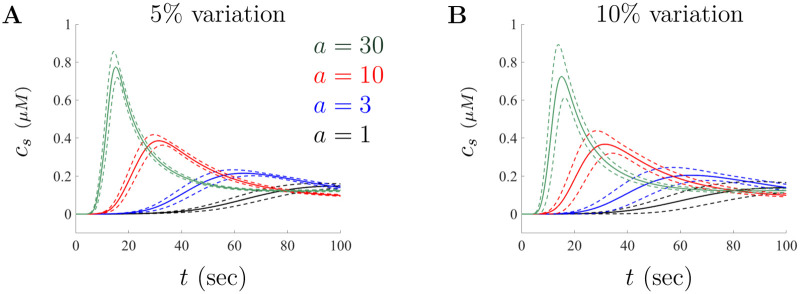
Model solutions under variation of parameters. The mean and corresponding standard deviations when the model is simulated for *n* = 100, 000 stochastically chosen parameter sets. The solid curves corresponds to the mean response and the dashed curves are the standard deviation above and below the mean. **A** and **B** illustrate the uncertainty in solutions when parameters are selected from a set with 5% and 10% deviation from the large doses base values given in Table 2, respectively.

To account for these uncertainty principles we vary the large doses parameters stochastically within 5% and 10% of baseline using a uniform distribution and generate *n* = 100, 000 solutions to the model. This type of simulation allows us to better understand the robustness of the model and provides some way to assess the influence of parameter selection on model results (see [49] for details on method). With the collection of *n* sample solution paths, we then compute the mean and standard deviation at each time *t*. Fig 9 shows the mean (solid curves) bounded within one standard deviation (dashed curves) for simulations around the concentration values *Aβ* = 3, 10, and 30, respectively. Again, we also include the result for *Aβ* = 1 for comparison and as a lower bound for the large doses range. The results illustrated in Fig 9 show that the model solutions are stable under parameter variation and continue to capture both the changes in amplitude and the peak time. Even if the large doses parameter set given in Table 2 is not optimal in minimizing our objective function, it does provide a reasonable set even under small perturbations. As such, our simulations convey evidence that the modeling assumptions may help capture how A*β* influences the cellular mechanisms involved in *PLC*-mediated IP_3_ production.

To better understand how each parameter impacts model solutions, we use sensitivity analysis based on partial rank correlation coefficients (PRCC). This allows us to determine the statistical relationship between model parameters and the resulting Ca^2+^ dynamics [50]. To do this, we characterize the resulting Ca^2+^ dynamics with two quantities: the peak Ca^2+^ concentration achieved during the simulation and the time at which this peak occurs. The PRCC measures the strength of the linear relationship between each model parameter and the model outcome after correcting for the linear effects of all other model parameters. The resulting PRCC scores take values between −1 and 1 with a negative value indicating that the model outcome decreases as the parameter increases and a positive value indicating that the model outcome increases as the parameter increases. The strength of the relationship between the model parameter and model output is indicated by the magnitude of the score.

The results of the PRCC are given in Tables 3 and 4. Table 3 shows the correlation to peak Ca^2+^ concentration while Table 4 shows the correlation of the time of peak. The tables list the correlation coefficients for each parameter when *a* = 3, 10, and 30. The ranking of the parameters was done by taking the average of the PRCC for the three doses of A*β*. As such, the parameters that most decrease the peak amplitude of Ca^2+^ solutions are the parameters *k*_*d*_, *k*_*b*_, and *K*_1_ while the parameters that most increase the amplitude are *V*_*Q*_, *k*_*c*_ and *k*_*a*_, as *a* is increased. Similarly, the parameters that most decrease the time of peak of Ca^2+^ solutions are the parameters *V*_*Q*_, *k*_*a*_, and *k*_*c*_ while the parameters that most increase the time peak are *K*_1_, *k*_*d*_ and *k*_*b*_. Although these parameters exhibit the strongest effect, we note that most other parameters exhibit a smaller but significant effect. Our intention is not to give a complete analysis for each model parameter, however we do analyze some interesting behaviors pertaining to specific parameters below.

**Table 3:** Partial rank correlation coefficient sensitivity analysis between model parameters (*n* = 100, 000) and the maximum Ca^2+^ concentrations for each of the three levels of A*β*. Results are with 10% variation in parameters values. * indicates the correlation coefficient is *not* significant at the *p* = 0.05 level.

Correlation to peak of signal			
	A*β* = 3	A*β* = 10	A*β* = 30
*k*_*d*_	-0.810	-0.796	-0.786
*k*_*b*_	-0.809	-0.796	-0.786
*K*_1_	-0.804	-0.793	-0.785
*k*_−2_	-0.767	-0.779	-0.772
*K*_*R*_	-0.647	-0.754	-0.781
*V*_*s*_	-0.631	-0.690	-0.738
*J*_*ER*_	-0.307	-0.259	-0.214
*K*_5_	-0.326	-0.206	-0.084
*V*_0_	-0.070	-0.076	-0.082
*K*_*PLC*_	-0.162	-0.021	0.053
*k*_5*p*_	-0.135	-0.021	0.042
*K*_3_	-0.027	-0.017	-0.010
*K*_*Q*_	0.002^*^	0.002^*^	0.001^*^
*k*_−4_	0.068	0.075	0.081
*K*_4_	0.161	0.173	0.180
*k*_*ip*3_	0.121	0.162	0.238
*δ*	0.461	0.155	0.002^*^
*K*_*s*_	0.712	0.514	-0.147
*γ*	0.636	0.693	0.735
*V*_*R*_	0.647	0.756	0.785
*k*_*f*_	0.751	0.773	0.788
*k*_3*k*_	0.795	0.774	0.751
*K*_2_	0.772	0.782	0.773
*k*_*a*_	0.810	0.797	0.788
*k*_*c*_	0.811	0.797	0.787
*V*_*Q*_	0.824	0.814	0.806

**Table 4:** Partial rank correlation coefficient sensitivity analysis between model parameters (*n* = 100, 000) and the time to peak when maximum Ca^2+^ concentration was reached for each of the three levels of large doses of A*β* concentration. ^*^ indicates the correlation coefficient is *not* significant at the *p* = 0.05 level.

Correlation to peak of signal			
	A*β* = 3	A*β* = 10	A*β* = 30
*V*_*Q*_	-0.832	-0.834	-0.838
*k*_*a*_	-0.827	-0.829	-0.831
*k*_*c*_	-0.827	-0.829	-0.831
*k*_3*k*_	-0.826	-0.828	-0.831
*V*_*R*_	-0.664	-0.782	-0.820
*K*_*s*_	-0.835	-0.773	-0.653
*γ*	-0.653	-0.647	-0.669
*k*_*f*_	-0.448	-0.482	-0.538
*J*_*ER*_	-0.331	-0.278	-0.242
*δ*	-0.494	-0.212	-0.056
*k*_−2_	-0.248	-0.229	-0.151
*K*_4_	-0.077	-0.082	-0.093
*k*_−4_	-0.028	-0.032	-0.039
*K*_3_	-0.012	-0.007	-0.003^*^
*K*_*Q*_	0.000^*^	0.000^*^	-0.000^*^
*k*_5*p*_	0.048	-0.004	-0.034
*k*_*ip*3*k*_	0.012	0.017	0.032
*V*_0_	0.030	0.032	0.037
*K*_5_	0.259	0.207	0.169
*K*_2_	0.261	0.238	0.156
*V*_*s*_	0.646	0.634	0.630
*K*_*PLC*_	0.727	0.662	0.607
*K*_*R*_	0.662	0.779	0.815
*k*_*b*_	0.826	0.825	0.819
*k*_*d*_	0.826	0.826	0.825
*K*_1_	0.829	0.830	0.831

When looking at the PRCC analysis, it appears that the PLC and G-protein rate constants *k*_*a*_, *k*_*b*_, *k*_*c*_, and *k*_*d*_ all have a large impact on the solution patterns in terms of solution peak and time to peak. Recall that, *k*_*a*_ and *k*_*c*_ are the activation rates for PLC and G-proteins, respectively. As the activation rates increase, this will lead to an increase in IP_3_ production and you will see the peak of the Ca^2+^ signal occur sooner. On the other hand, *k*_*b*_ and *k*_*d*_ correspond to the inactivation of PLC and G-proteins, respectively. A higher inactivation rate for both PLC and G-proteins will decrease IP_3_ production and thus lower the peak amplitude of Ca^2+^ responses. From a biological perspective this makes sense, once PLC is activated, the production of IP_3_ occurs through hydrolysis of phosphatidylinositol-4,5-biphosphate (PIP2). Thus, as the amount of active PLC increases, we should see an increase in the amplitude peak and a decrease in the time to peak in Ca^2+^ responses. Conversely, as the amount of active PLC decreases, we should see a decrease in the amplitude peak but an increase in the time to peak in Ca^2+^ responses as fewer IP_3_ are available for binding to the IP_3_R. Even though these results align with what one might suspect occurs from a biological perspective, these behaviors are directly linked to how the model was constructed. Specifically, recall that the subsystem given by Eqs (15)–(17) is solely driven by *PLC* and A*β*. Changes in PLC will play a major role in the amount of IP_3_ available for IP_3_R binding. Further analysis on the impact of these parameters is provided below.

As noted above, the parameter *K*_1_ also plays a major role in solution patterns. As adapted from the De Young and Keizer (1992) model, this parameter corresponds to the effective binding rate of IP_3_ to one of the IP_3_R model subunits when no inactivating Ca^2+^ is present. As such, this parameter helps drive the IP_3_R dynamics. In the model, an increase in *K*_1_ has an inactivating effect on the IP_3_R since either the unbinding rate of IP_3_ to receptor binding site is increased or the binding rate is decreased. In either case, this would decrease the opportunity for the receptor to remain in an active and open state. The PRCC analysis highlights that *K*_1_ is critical for understanding the Ca^2+^ patterns of the model. Because of the influence of this parameter on model solutions, this suggests that the IP_3_R dynamics does contribute to the observed Ca^2+^ patterns. We analyze the model below to further expand on the influence of *K*_1_ on model solutions. As the model suggests that changes in *K*_1_ may be dependent on A*β* levels, further investigations on the connection between the IP_3_R and A*β* may be necessary.

The PRCC also highlights additional interesting information regarding the influence of specific parameters on model solutions. Interestingly, the dependence of solution amplitude peak with respect to the parameter *K*_*s*_ appears to be tied with the size of *a*. More specifically, the PRCC for *K*_*s*_ when *a* = 3, 10, and 30 are 0.712, 0.514, and −0.147, respectively. This implies that as *a* increases, altering *K*_*s*_ has a different effect on model amplitude. Namely, the amplitude increases for *a* = 3 and *a* = 10, but decreases when *a* = 30. Notice that similar results occur for the parameters *K*_*PLC*_ and *k*_5*p*_ but in the opposite direction. The dependence of solution time to peak with respect to the parameter *δ* also appears to be linked to the value of *a*. In this case, the PRCC for *δ* when *a* = 3, 10, and 30 are −0.494, −0.212, and −0.056, respectively. Although the sign of the PRCC is negative in each case, the disparity of the correlation coefficient may indicate that A*β* is affecting the intrinsic background production of active *G*-proteins differently as the doses vary. The dependence of *a* on these parameters suggests that A*β* is impacting the mechanisms differently as the amount of A*β* is altered. Further exploration of these parameters may tease out additional information about the influence of A*β* on cellular mechanisms but is beyond the scope of this study.

### 4.2 Impact of A*β* on IP_3_R for large doses

The impact of A*β* on the IP_3_ signaling cascade appears to be concentration dependent. Not surprising, the PRCC analysis suggests that the rates *k*_*a*_, *k*_*b*_, *k*_*c*_, and *k*_*d*_ play a significant role on the amplitude of responses and the peak time. These parameters directly influence the amount of *PLC* that feeds into the subsystem given by Eqs (15)–(17) and small variations in these parameters will greatly affect the solutions of the model. Instead of looking specifically at these parameters, we can alternatively investigate the impact of changes in *V*_*Q*_. Recall that *V*_*Q*_ controls the influence of A*β* on PLC-mediated IP_3_ production. As such, it is no surprise that *V*_*Q*_ also plays a significant role in the solution patterns.

**Fig 10:**
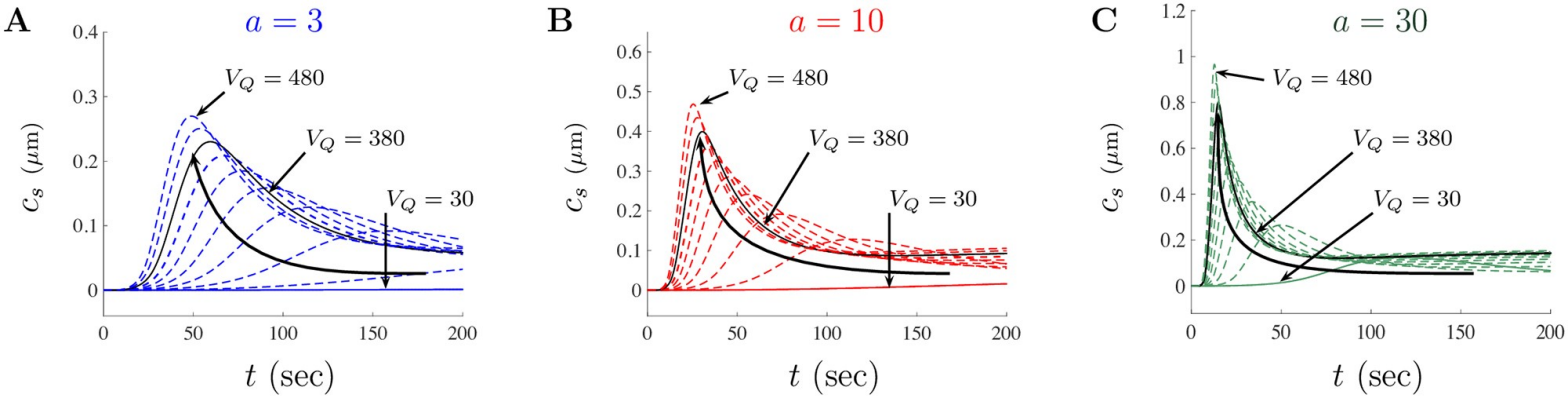
PRCC prediction on solution amplitude and time to peak for model parameter *V*_*Q*_. The impact of *V*_*Q*_ is shown as a series of curves for *a* = 3, *a* = 10, and *a* = 30 in **A**, **B**, and **C**, respectively. In each diagram, the curved black arrow tracks the shift in the peak of solutions as *V*_*Q*_ takes on various values ranging from *V*_*Q*_ = 30 to *V*_*Q*_ = 480. The black trace in each diagram represents the baseline *V*_*Q*_ value for the large doses parameter set.

Fig 10 shows the impact of altering *V*_*Q*_ on model solutions for *a* = 3, 10, and 30, in A, B, and C, respectively. As the parameter *V*_*Q*_ increases from the small doses value of *V*_*Q*_ = 7.82 to the large doses value of *V*_*Q*_ = 380 we see that the model solutions shift up and to the left. This is highlighted by the curved arrow in each figure. The large doses value of *V*_*Q*_ = 380 has been singled out using the solid black trace while 9 other solutions (with various *V*_*Q*_ values) are shown as dashed colored traces. The solution for the values *V*_*Q*_ = 30, 380, and 480 have been highlighted in each figure for reference. The results of Fig 10 also confirm the PRCC analysis that *V*_*Q*_ is positively correlated with the peak amplitude and negatively correlated with the peak time onset. Clearly, altering *V*_*Q*_ impacts both the solution amplitude and the time to peak.

Although the PRCC identifies the parameter *K*_1_ for example, as playing a significant role on model solution’s amplitude and time to peak, the PRCC analysis cannot capture how variations in a single parameter will affect model solutions in general. For example, it is not evident in the PRCC analysis that the parameter *k*_−4_ plays a significant role on solutions and is a critical parameter when considering the large doses Ca^2+^ signaling patterns observed experimentally. Varying *k*_−4_ has a direct impact on the Ca^2+^ signal tail and partly controls the decay of the signals, but does not alter the amplitude or time to peak significantly. Both *K*_1_ and *k*_−4_ are parameters that help control the dynamics of IP_3_Rs.

**Fig 11:**
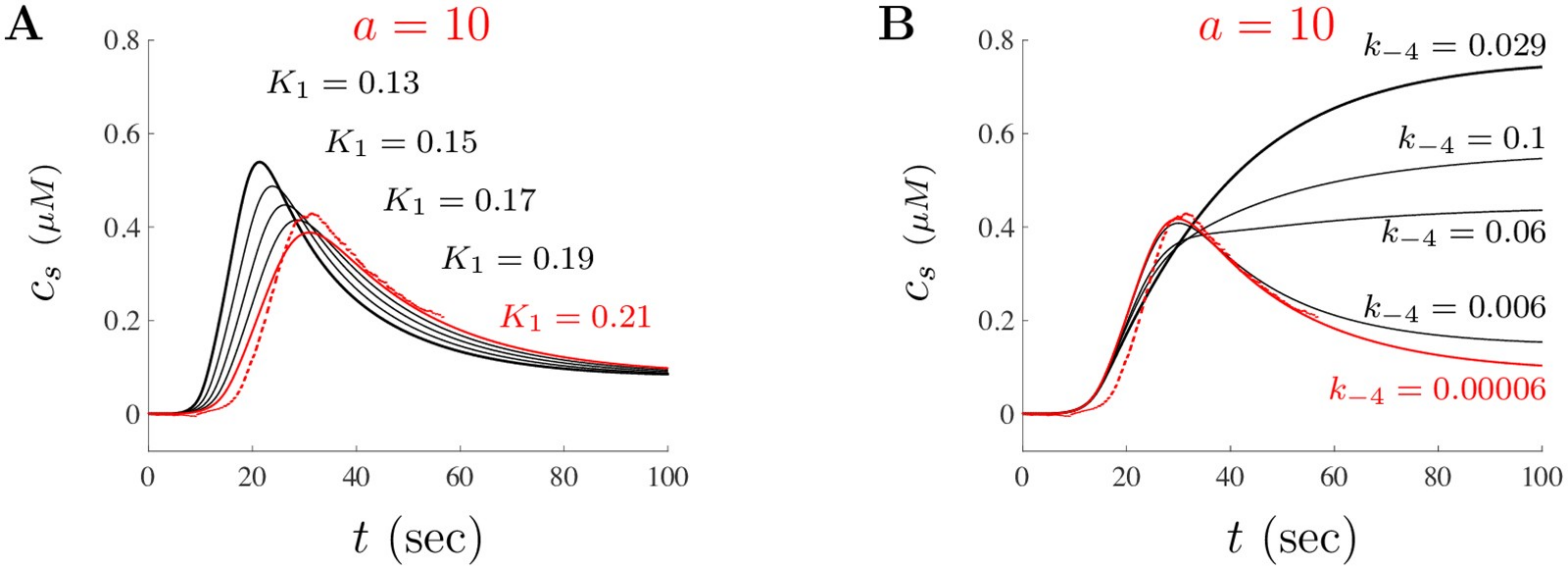
Impact of large doses of A*β* on IP_3_R dynamics. The impact of the IP_3_R model parameters *K*_1_ and *k*_−4_ are shown in **A** and **B**, respectively. The traces shown use the large doses parameters except for the values highlighted in each diagram. The top bold black traces correspond to the model solution when the parameter value for the small doses is used. The red traces are the model solutions for the parameter values of the large doses. The black traces between the bold and red correspond to intermediate parameter values as given in each diagram.

Shown in Fig 11 are two diagrams that illustrate the impact of A*β* on the IP_3_R itself through the parameters *K*_1_ and *k*_−4_ when *a* = 10 (a similar effect occurs for *a* = 3 and *a* = 30). Fig 11A shows the representation of the effects of varying *K*_1_ model solutions. Starting with the large doses parameters, we simulate the model by altering *K*_1_ from the base small doses value of *K*_1_ = 0.13 (bold black trace) and increasing the parameter to the large doses value *K*_1_ = 0.13 (smooth red trace). As is suggested by the PRCC analysis, we see that *K*_1_ is negatively associated with the peak amplitude and positively correlated with respect to the time to peak. The impact of changes to the parameter *k*_−4_ is shown in Fig 11B. Similar to Fig 11A, starting with the small doses parameter value *k*_−4_ = 0.029 (bold black trace) and decreasing the parameter to the large doses value *k*_−4_ = 0.00006 (red trace) shows that *k*_−4_ plays a critical role in controlling the decay of Ca^2+^ signals. Interestingly, the PRCC does not capture this effect as it was only conducted to track the impact on the amplitude peak and latency of solutions. Altering the other IP_3_R parameters will have various effects on solutions similar to the impact of varying *K*_1_. Changes to IP_3_R parameters seem necessary in order to capture the increasingly rapid decay and suggests that A*β* for large doses may act to desensitize the IP_3_R.

Whether A*β* directly interferes with IP_3_Rs remains debatable but our model suggests that A*β* does indeed alter the receptor dynamics for large doses. There may be some intrinsic threshold on A*β* concentration within the cellular environment for which the sensitivity of IP_3_Rs is affected by A*β*. Of particular interest is the role of the IP_3_R parameters in capturing the observed rapid decay of Ca^2+^ signal for large A*β* doses.

### 4.3 Limitations of the model

As with any mathematical model, many limitations exist with the approach presented here. Because of our interest in dissecting the effects of A*β* on the IP_3_ signaling cascade, the model development and construction utilized a number of simplifying assumptions. While many of these assumptions are traditional, the simplistic nature of the model cannot fully represent the biological environment in a holistic way. None-the-less, our approach has sought to balance the complex biophysical mechanisms involved in Ca^2+^ signaling with that of a mathematical structure that can be useful in identifying key factors involved in generating certain solution patterns. Unfortunately, a lack of data has made it difficult to determine the precise conditions and the validity of many of the modeling assumptions. For example, we acknowledge that the steady-state assumptions and the particular mechanisms for how A*β* may be interfering in the Ca^2+^ signaling process need to be explored further. Although these assumptions contributed to model solutions whose behavior and dynamics match experimental results, more data is needed to fully justify these assumptions. Additionally, the inclusion of other Ca^2+^ regulatory mechanisms will be necessary to describe whole-cell calcium dynamics in a biologically robust way.

Our model construction assumes that iA*β*_42_Os (1) act as an agonist for G-protein activation, and (2) affect the maximal rate of PLC mediated IP_3_ production. The second assumption was developed based on the results of a series of Monte Carlo numerical simulations that considered a wide-array of possible sites for including the impact of iA*β*_42_Os on cellular mechanisms. These simulations were conducted using a large number of initial parameter sets and a variety of functional representations (such as Hill functions of various degrees). Although we were able to match some of the observed experimental results for large doses without including the assumption given in Eq (14), we could not reproduce the three Ca^2+^ signals (*a* = 3, 10, and 30) with the same parameter set simultaneously. Furthermore, any parameter set that closely matched the changes in amplitude and time to peak for small doses of *a* could not reproduce any spiking behavior observed through cellular and SERCA parameter variations unless *V*_*Q*_ ≠ 0. That led us to incorporate the A*β*-dependent term for the maximal rate of PLC mediated IP_3_ production given in Eq (14). Due to the complex dependence on model parameters, it may be that this model assumption does not accurately capture how A*β* interferes with the IP_3_ production pathway. However, it proved valuable in reproducing observed data for both the small and large doses and provides a possible avenue for further investigations.

As with any model involving numerous parameters, solutions will vary based on the parameter set utilized. In this work, we first rescaled the experimental data, then fitted our model using a best fit parameter estimation procedure. When alternative scaling parameters are used, the model parameters will necessarily change. However, our results show that the model captures the changes in the amplitude and peak time of the signals in a robust and predictable way for both small and large doses of iA*β*_42_Os. The PRCC analysis also provides a structured way for understanding how each individual parameter impacts model solutions. Further analysis of our PRCC results could bring to light additional parameter and A*β*-related dependencies. For example, the PRCC values for some parameters are highly dependent on A*β* concentration. Such parameters may also play an important role in determining the possible kinetic interaction of A*β* within the IP_3_ production cascade.

## 5 Discussion

Ca^2+^ is one of the most versatile and universal signals in the human body playing a pivotal role in controlling numerous aspects in the physiology and biochemistry of neurons [51]. Accordingly, intracellular Ca^2+^ dysregulation has been implicated in a wide variety of immunological disorders and neurodegenerative diseases including Alzheimer’s, Parkinson’s, and Huntington’s disease. In neurons, as in many other cell types, IP_3_-mediated elementary Ca^2+^ signals, also referred to as puffs, are the building blocks of cellular Ca^2+^ signaling, and arise through the concerted opening of clustered IP_3_Rs coordinated via a Ca^2+^-induced Ca^2+^-release mechanism [52]. Although the cytosolic Ca^2+^ dependency of IP_3_Rs has been well characterized, little is known as to how changes in basal cytosolic [Ca^2+^] would alter the dynamics of IP_3_-evoked Ca^2+^ signals in disease cells, such as neuronal cells of Alzheimer’s and Parkinson’s disease brains. In AD, iA*β*Os are now believed to play a major role in the early phase of the disease as their intracellular rise correlates well with the symptoms of AD [3, 53]. More generally, A*β*Os have been found to be predictive of cognitive status at death among patients with AD [54]. Various mechanisms have been proposed to correlate the progressive intracellular Ca^2+^ elevation with the concomitant increase of iA*β*Os observed in neurons during the progression of the AD [25]. Among them, the detrimental activity of iA*β*Os on the normal functioning of the IP_3_-signaling pathway has been indicated as a potential mechanism responsible for alteration of the Ca^2+^ homeostasis in AD neurons.

We and others have suggested that a G-protein mediated activation of PLC by iA*β*_42_Os is responsible for the overproduction of IP_3_ and consequent rise of cytosolic Ca^2+^ observed in cells exposed to iA*β*_42_Os [14, 18]. Moreover, others have suggested that A*β* may cause cytosolic Ca^2+^ rise by a mixed mechanisms of PLC-dependent and independent manner [15, 16, 55]. The effect of iA*β*_42_Os on intracellular Ca^2+^ fluxes have previously been investigated by developing a computational model to study important intracellular Ca^2+^ pathways in normal and in iA*β*_42_Os affected conditions [27]. However, no upstream IP_3_ production processes were incorporated in the model. Here, we have illustrated a possible mechanistic way for how iA*β*_42_Os triggers IP_3_ overproduction with consequent rise in cytosolic Ca^2+^ by including some mechanisms of upstream IP_3_ production in the model. Specifically, we pinpoint two main possible sites of action for iA*β*_42_Os to interact in the cascade of events resulting from stimulation of G-protein in the plasma membrane to the release of Ca^2+^ from the ER.

In our previous study [18], we argued that it was unlikely that iA*β*_42_Os act on IP_3_Rs in the generation of A*β*-induced Ca^2+^ signaling events. The results of the model are consistent with this for the small doses parameters. However, the model also suggests that iA*β*Os may in-fact be directly affecting the IP_3_Rs when large doses are introduced. The analysis illustrated in Fig 11 helps us understand what happens to Ca^2+^ signaling in the presence of iA*β*_42_Os as changes to IP_3_Rs occur. The persistent increase of iA*β*_42_Os may alter the sensitivity of IP_3_Rs to Ca^2+^ over time. For large doses of iA*β*_42_Os, IP_3_Rs may become more sensitive to low- or sub-threshold IP_3_ levels and in turn trigger local and global Ca^2+^ signaling events. The fact that the parameter *k*_−4_ appears to play a major role in the decay of observed Ca^2+^ signals singles out the potential that iA*β*_42_Os do act on the IP_3_R itself, at least for large doses. Our model suggests the need for further investigation on the relationship between iA*β*_42_Os and the sensitivity of IP_3_Rs to IP_3_ levels.

Our approach provides a precise way to incorporate the effects of iA*β*_42_Os on IP_3_ signaling mechanisms that does not necessarily depend on the choice of the IP_3_R model. When a saturating binding rate model for the IP_3_R model is used (as that used in [33] instead of the Li-Rinzel formulation), such a model can also capture the changes in amplitude and peak times for large doses using the same upstream modeling assumptions as outlined above (unpublished results J. Latulippe). This provides further justification that the modeling kinetics of the possible interactions of iA*β*_42_Os with G proteins and PLC may be sufficiently captured by the model. Additionally, Toglia et al. [27] have also suggested a relationship between IP_3_ concentration and iA*β*_42_Os. However, their investigation assume that IP_3_ concentration levels are impacted by iA*β*_42_Os but use a data fitting procedure to do this rather than attributing those changes to upstream mechanisms. As such, we believe that the model presented here is the first to quantify possible mechanisms for how iA*β*_42_Os affects the upstream mechanisms in the IP_3_ signaling cascade.

Although our model considers the impact of iA*β*_42_Os specifically on the IP_3_ signaling cascade in oocytes, our results could be useful in more complex models of various cells. Existing astrocyte models (such as [34, 56–58]) that incorporate Ca^2+^ dynamics could be altered to include the effects of A*β* on IP_3_ signaling components described in this study. This would provide a way to test model assumptions and determine whether solution patterns are consistent in different model environments. Furthermore, the current model could be expanded to include additional pumps and channels known to play a role in various cell types. Incorporating data driven models within the Ca^2+^ modeling toolbox may prove to be an efficient way to develop whole cell models that can be used to study how A*β* alters various signaling pathways. For example, the ability to express exogenous proteins, including NMDA Receptors, provides a powerful tool as a possible next step in developing increasingly elaborate mathematical models capable of more closely mimicking neuronal behavior.

Because of the complex cross-talk nature of Ca^2+^ signaling, our model also provides a way to control for and test various therapeutic strategies in a modeling environment. For example, to mimic the intrinsically slow accumulation of A*β* seen in the pathology of AD, A*β* can be introduced very slowly into the model and solutions simulated accordingly. We can then introduce artificial agonists or antagonists that affect G-protein activation and PLC function to see how they affect Ca^2+^ signals over various timescales. Using the model to better understand what happens to Ca^2+^ regulation in these simulations can directly influence and suggest how one could control Ca^2+^ signaling in the presence of A*β*, and more generally, various AD environments.

The results of this study suggest the need for two different dose-dependent models to incorporate changes in cellular Ca^2+^ signaling in the presence of increasing concentrations of iA*β*_42_Os. In *in vivo* environments, it may be the case that in the early phase of AD, slowly accumulating levels of iA*β*Os remain relatively small. Under such conditions, the small doses model may be better suited than the large doses model. Regardless, our model development and analysis suggests that increasing the amount of iA*β*_42_Os present in the cell can have a pervasive impact on numerous cellular mechanisms.

Building computational models can help provide a better understanding for the complex cross-talk between various signaling mechanisms within neurons, something difficult to establish with current experimental capabilities. Through further analysis and development, researchers can use the model to formulate novel experimental procedures and eventually suggest new therapies for treating AD.

## Appendix

According to Maravall et al. (2000) [28], changes in Ca^2+^ concentration are associated with changes in fluorescence through the equation

$$\begin{array}{c} \frac{c-c_{0}}{K_{D}}=f_{m}\left(1-1/R_{f}\right)\frac{\delta f}{\left(\delta f_{max}-\delta f\right)\delta f_{max}}, \end{array}$$ (23)

where *K*_*D*_ is a dissociation constant, *f*_*max*_ is the intensity of the dye at maximum Ca^2+^ concentration, *R*_*f*_ = *f*_*max*_/*f*_*min*_ is the indicator’s dynamic range with *f*_*min*_ being the intensity at minimum Ca^2+^ concentration, *δf*_*max*_ is the saturation of the Ca^2+^ indicator, *f*_*m*_ = *f*_*max*_/*f*_0_, and *c*_0_ is the resting Ca^2+^ concentration. The values of *K*_*D*_ and *R*_*f*_ are associated with attributes of the indicator in a particular cellular environment [28] and as such are independent of cellular properties. Wavelength ratio measurements do not generally depend on dye concentration, optical path length, excitation intensity, or detector efficiency [28]. However, the value of *K*_*D*_ and the dynamic range of *R*_*f*_ may vary batch to batch and should be estimated using a similar protocol and cellular cytoplasmic domain [28, 59].

In Eq (23), *δf*_*max*_ is the key parameter needed for establishing the conversion from fluorescence to Ca^2+^ concentrations. When we fix the initial Ca^2+^ concentration, *c*_0_, we can estimate *δf*_*max*_ using

$$\begin{array}{c} \delta f_{max}=\frac{\left(1-1/R_{f}\right)}{1/R_{f}+c_{0}/K_{D}}, \end{array}$$ (24)

as long as true saturation is attained [28] and *K*_*D*_ and *R*_*f*_ are known. In practice, *δf*_*max*_ can be used to estimate the unknown resting Ca^2+^ concentration by inverting the relationship in Eq (24).

**Fig 12:**
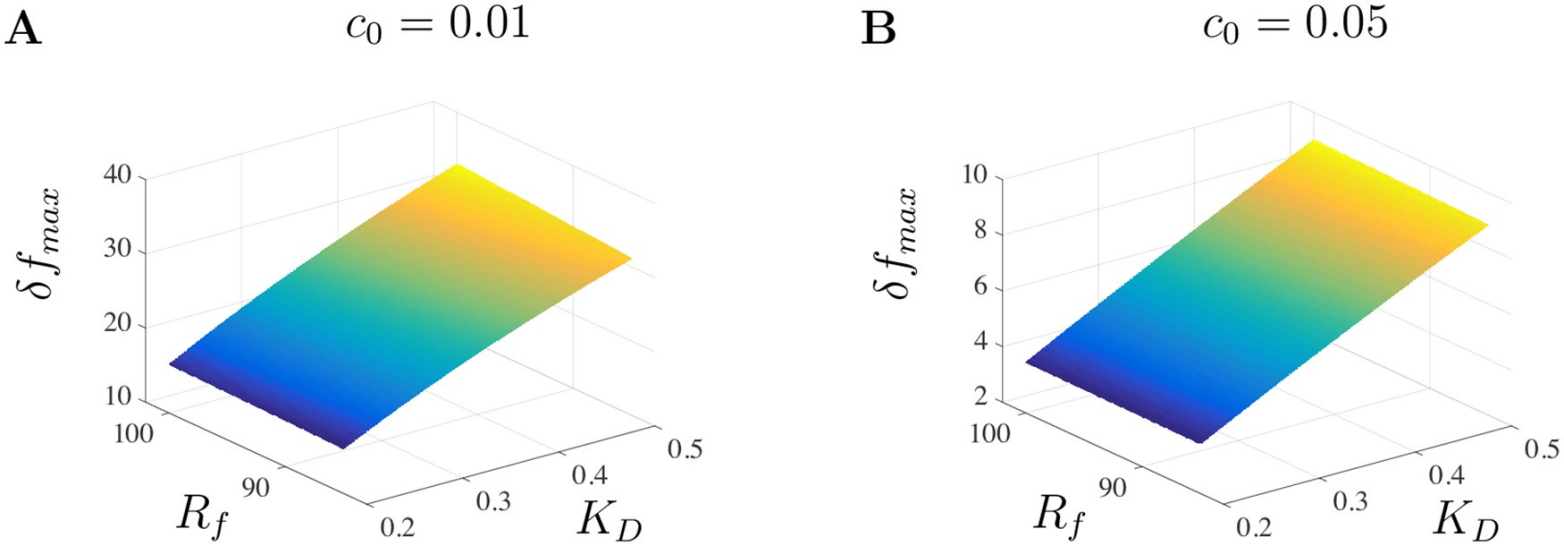
Uncertainty in estimation of *R*_*f*_ have minimal effect on data rescaling. Effects of *K*_*D*_ and *R*_*f*_ on *δf*_*max*_ under initial Ca^2+^ concentration *c*_0_ = 0.01 in **A** and *c*_0_ = 0.05 in **B**. Notice that *R*_*f*_ has minimal effect on *δf*_*max*_ while *K*_*D*_ alters the value of *δf*_*max*_.

With Eqs 23 and 24 in hand, converting fluorescence data to Ca^2+^ concentrations only requires obtaining values for *f*_*m*_, *R*_*f*_, and *δf*_*max*_ during experimental procedure. However, these values are often not reported in favor of the traditional *δf* fluorescence measurements and extracting them from reported changes in fluorescence ratio, or establishing their values *a posteriori*, can be challenging. As such, in order to complete a conversion for data given in terms of *δf* we approximate a number of parameters. Since both *K*_*D*_ and *R*_*f*_ depend on indicator properties, they can be approximated for a variety of indicators. Based on the experiment in [18], we assume values of *K*_*D*_ ≈ 0.2−0.5 *μ*M and that *R*_*f*_ has a dynamic range *R*_*f*_ ≈ 85−100 and note that uncertainties in *R*_*f*_ have minimal affect on Eq (24). We illustrate this in Fig 12 where *δf*_*max*_ is plotted as a function of *K*_*D*_ and *R*_*f*_ when *c*_0_ = 0.01 *μ*M and *c*_0_ = 0.05 *μ*M in **A** and **B**, respectively.

As can be seen from Fig 12, *R*_*f*_ has little effect on the value of *δf*_*max*_. This is consistent with the idea that for indicators with a large dynamic range, the exact value of *R*_*f*_ is insignificant [28]. For indicators such as Fluo 4, *K*_*D*_ is often assumed to be between 0.25 and 0.5 *μ*M but some studies suggest that *K*_*D*_ may have much greater range [60, 61].

**Fig 13:**
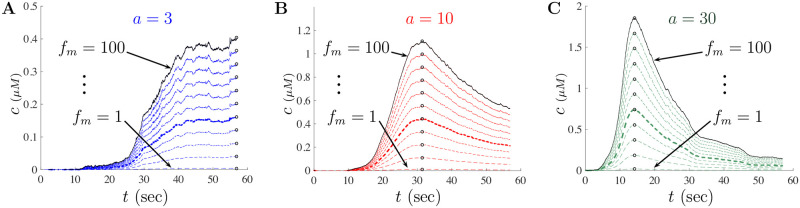
Impact of *f*_*m*_ on rescaling of Ca^2+^ data. Changes in scaled experimental data when *f*_*m*_ ranges from 1 to 100. In all figures, *R*_*f*_ = 100, *KD* = 0.3, and *c*_0_ = 0.05. Figs **A**, **B**, and, **C** correspond to the scaled data for *a* = 3, *a* = 10, and *a* = 30, respectively. The maximum value of each scaled experimental data set is shown by the open circle. The bold color curve corresponds to *f*_*m*_ = 40, the value used throughout the simulations.

Without loss of generality, here we consider a basal Ca^2+^ concentration of *c*_0_ = 0.05 *μ*M and set *K*_*D*_ = 0.3 *μ*M and *R*_*f*_ = 100. Because we have no previous knowledge for the value of *f*_*m*_, we consider a range *f*_*m*_ ≈ 1−100 where the exact value depends on the ratio of the maximal intensity and the resting intensity. Using these values, we plot the corresponding Ca^2+^ concentrations from the fluorescence data in [18] for various estimates of *f*_*m*_. Fig 13A–13C show the time traces of the converted fluorescence data for the impact of a 10 nl injection of A*β* at concentrations of *a* = 3 *μ*g/ml, *a* = 10 *μ*g/ml, and *a* = 30 *μ*g/ml, respectively. In Fig 13A–13C each dashed plot corresponds to a different value of *f*_*m*_ ranging from *f*_*m*_ = 1 to *f*_*m*_ = 100 (black) with *n* = 11 (*f*_*m*_ = 1, 10, 20, …, 100). The maximum value is also highlighted for each conversion plot (circle) and provides the peak time for the three A*β* levels.

**Fig 14:**
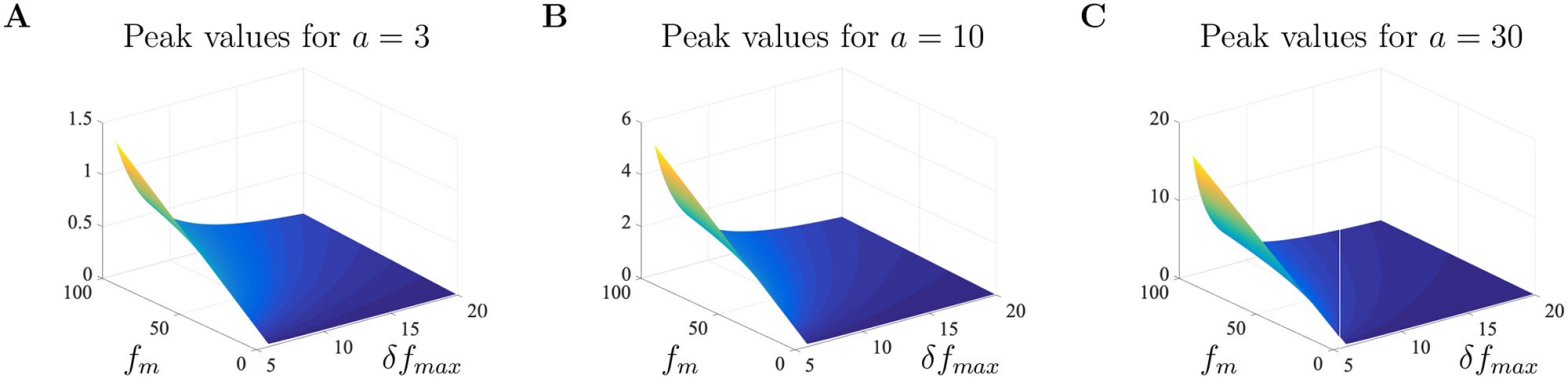
Peak values as a function of scaling parameters. Peak values of the data conversion as a function of *f*_*m*_ and *δf*_*max*_ for *a* = 3 **A**, *a* = 10 **B**, and *a* = 30 **C**. In all simulations, *R*_*f*_ = 100, *KD* = 0.3, and *c*_0_ = 0.05 were fixed. This figure shows that the greatest scaling affects occur when *f*_*m*_ and *δf*_*max*_ are large and small, respectively.

To study the impact of the conversion to Ca^2+^ concentrations, Fig 14A–14C shows the corresponding maximum value of the concentration as a function of *f*_*m*_ and the range of *δf*_*max*_ between 6 and 20. These three dimensional plots allow us to better understand the impact of the conversion parameters on the maximum values of the fluorescence data in [18]. Again, because we do not have estimates for *f*_*m*_ or *c*_0_, a true conversion from fluorescence to concentration is elusive. However, in all the profiles illustrated, each conversion does capture the changes in amplitude and latency to peak time observed experimentally as levels of A*β* are increased.
